# Supplementary material for: Thunder-DDA-PASEF enables high-coverage immunopeptidomics and is boosted by MS2Rescore with MS2PIP timsTOF fragmentation prediction model
Source: Nat Commun. 2024 Mar 13;15:2288. doi: 10.1038/s41467-024-46380-y (PMC10937930; doi:10.1038/s41467-024-46380-y)
Supplement: Supplementary file 1 — Supplementary Information [file 41467_2024_46380_MOESM1_ESM.pdf]

# Thunder-DDA-PASEF enables high-coverage immunopeptidomics and is boosted by MS$^2$Rescore with MS$^2$PIP timsTOF fragmentation prediction model

David Gomez-Zepeda[1,2,3*], Danielle Arnold-Schild[1], Julian Beyrle[1,2,3], Arthur Declercq[4,5], Ralf Gabriels[4,5], Elena Kumm[1], Annica Preikschat[1], Mateusz Krzysztof Łącki[1], Aurélie Hirschler[6], Jeewan Babu Rijal[6], Christine Carapito[6], Lennart Martens[4,5], Ute Distler[1,7], Hansjörg Schild[1,7], Stefan Tenzer[1,2,3,7*]

[1]Institute of Immunology, University Medical Center of the Johannes-Gutenberg University, Mainz, Germany.

[2]Helmholtz Institute for Translational Oncology Mainz (HI-TRON Mainz) - A Helmholtz Institute of the DKFZ, Mainz, Germany

[3]German Cancer Research Center (DKFZ) Heidelberg, Division 191, Germany

[4] VIB-UGent Center for Medical Biotechnology, VIB, Ghent, Belgium

[5] Department of Biomolecular Medicine, Ghent University, Ghent, Belgium

[6] BioOrganic Mass Spectrometry Laboratory (LSMBO), IPHC UMR 7178, University of Strasbourg, CNRS, ProFI - FR2048, Strasbourg, France

[7]Research Center for Immunotherapy (FZI), University Medical Center of the Johannes-Gutenberg University, Mainz, Germany

*To whom correspondence should be addressed.

19/February/2024

This document contains supplementary text, methods, tables and figures. The table of content is clickable and the links direct to the sections and subsections in this document. A description of the Supplementary Data files can be found in the Inventory of Supporting Information.

# Contents


**1 Supplementary Methods** **3**

1.1 SARS-CoV-2 protein sequence fragments coded to by expressed by JY and Raji transfectants . . 3

**2 Supplementary Notes** **4**

2.1 Restricting the TIMS range results in the loss of singly charged peptides . . . . . . . . . . . . . . 4

**3 Supplementary Figures** **5**

3.1 Fig. S1. Restricting the TIMS range results in the loss of singly charged peptides . . . . . . . . . 5

3.2 Fig. S2. The HLAIp-tailored isolation polygon results in fewer long peptides (> 13 AAs) . . . . 6

3.3 Fig. S3. Peptide distribution across the $1/K_0$ vs m/z dimensions for multiple samples acquired without or with the Thunder isolation polygon . . . . . . . . . . . . . . . . . . . . . . . . . . . 7

3.4 Fig. S4. HLAIp distribution across the $1/K_0$ vs m/z dimensions for multiple samples acquired without or with the Thunder isolation polygon . . . . . . . . . . . . . . . . . . . . . . . . . . . 8

3.5 Fig. S5. Dynamic range in 100 ms and 300 ms methods without and with the Thunder isolation polygon for multiple samples . . . . . . . . . . . . . . . . . . . . . . . . . . . . . . . . . . . . 9

3.6 Fig. S6. HLAIp sequence motifs of the 9-mers dentified in JY, HeLa, SK-MEL-37, and plasma . 10

3.7 Fig. S7. HLA-specific HLAIp distribution across the $1/K_0$ vs m/z dimensions for multiple samples acquired without or with the Thunder isolation polygon . . . . . . . . . . . . . . . . . 11

3.8 Fig. S8. Charge distribution in function of the HLA allotype . . . . . . . . . . . . . . . . . . . . 12

3.9 Fig. S9. Evaluation of the timsTOF prediction models . . . . . . . . . . . . . . . . . . . . . . . . 13

3.10 Fig. S10. Peptide and protein overlap between biological replicates of JY and Raji . . . . . . . . 14

3.11 Fig. S11. Peptide characteristics of HLAIps in the JY and Raji spike-transfected experiment by allotype . . . . . . . . . . . . . . . . . . . . . . . . . . . . . . . . . . . . . . . . . . . . . . . 15

3.12 Fig. S12. Hierarchical structure visualization of the GO enrichment analysis of JY Raji common proteins covered by HLA class I ligands . . . . . . . . . . . . . . . . . . . . . . . . . . . . . 16

3.13 Fig. S13. Spike immunopeptides fragmentation spectra comparison vs. synthetic or predicted . . 20

# 1 Supplementary Methods

## 1.1 SARS-CoV-2 protein sequence fragments coded to by expressed by JY and Raji transfectants

The pcDNA3.1-SARS2-Spike vector containing the full-length cDNA encoding for the SARS-CoV2 Spike protein was obtained from Fang Li (Addgene plasmid 145032 ; https://www.addgene.org/145032/) [1]. The Spike S cDNA was split into S1 (2016 bp) and S2 (1761 bp) subunits for cloning by PCR into the NheI and XhoI restriction sites from the multiple cloning site of the pcDNA3.1+P2AeGFP vector (Genscript). The corresponding spike protein sequence is shown below, with the fragments indicated in blue for S1 and red for S2.

>sp|P0DTC2|SPIKE_SARS2

1 MFVFLVLLPLVSSQCVNLTTRTQLPPAYTNSFTRGVYYPDKVFRSSVLHSTQDLFLPF 58

59 FSNVTWFHAIHVSGTNGTKRFDNPVLPFNDGVYFASTEKSNIIRGWIFGTTLDSKTQS 116

117 LLIVNNATNVVIKVCEFQFCNDPFLGVYYHKNNKSWMESEFRVYSSANNCTFEYVSQP 174

175 FLMDLEGKQGNFKNLREFVFKNIDGYFKIYSKHTPINLVRDLPQGFSALEPLVDLPIG 232

233 INITRFQTLLALHRSYLTPGDSSSGWTAGAAAYYVGYLQPRTFLLKYNENGTITDAVD 290

291 CALDPLSETKCTLKSFTVEKGIYQTSNFRVQPTESIVRFPNITNLCPFGEVFNATRFA 348

349 SVYAWNRKRISNCVADYSVLYNSASFSTFKCYGVSPTKLNDLCFTNVYADSFVIRGDE 406

407 VRQIAPGQTGKIADYNYKLPDDFTGCVIAWNSNNLDSKVGGNYNYLYRLFRKSNLKPF 464

465 ERDISTEIYQAGSTPCNGVEGFNCYFPLQSYGFQPTNGVGYQPYRVVVLSFELLHAPA 522

523 TVCGPKKSTNLVKNKCVNFNFNGLTGTGVLTESNKKFLPFQQFGRDIADTTDAVRDPQ 580

581 TLEILDITPCSFGGVSVITPGTNTSNQVAVLYQDVNCTEVPVAIHADQLTPTWRVYST 638

639 GSNVFQTRAGCLIGAEHVNNSYECDIPIGAGICASYQTQTNSPRRARSVASQSIIAYT 696

697 MSLGAENSVAYSNNSIAIPTNFTISVTTEILPVSMTKTSVDCTMYICGDSTECSNLLL 754

755 QYGSFCTQLNRALTGIAVEQDKNTQEVFAQVKQIYKTPPIKDFGGFNFSQILPDPSKP 812

813 SKRSFIEDLLFNKVTLADAGFIKQYGDCLGDIAARDLICAQKFNGLTVLPPLLTDEMI 870

871 AQYTSALLAGTITSGWTFGAGAALQIPFAMQMAYRFNGIGVTQNVLYENQKLIANQFN 928

929 SAIGKIQDSLSSTASALGKLQDVVNQNAQALNTLVKQLSSNFGAISSVLNDILSRLDK 986

987 VEAEVQIDRLITGRLQSLQTYVTQQLIRAAEIRASANLAATKMSECVLGQSKRVDFCG 1044

1045 KGYHLMSFPQSAPHGVVFLHVTYVPAQEKNFTTAPAICHDGKAHFPREGVFVSNGTHW 1102

1103 FVTQRNFYEPQIITTDNTFVSGNCDVVIGIVNNTVYDPLQPELDSFKEELDKYFKNHT 1160

1161 SPDVDLGDISGINASVVNIQKEIDRLNEVAKNLNESLIDLQELGKYEQYIKWPWYIWL 1218

1219 GFIAGLIAIVMVTIMLCCMTSCCSCLKGCCSCGSCCKFDEDDSEPVLKGVKLHYT 1273

# 2 Supplementary Notes

## 2.1 Restricting the TIMS range results in the loss of singly charged peptides

Feola *et al.* [2] used a microfluidics system (PeptiCHIP) to enrich HLAIps from JY cells starting from 50, 10, or 1 million cells, in duplicates of sample preparation. Then, they injected all the peptides from each sample in nanoLC-MS using a nanoElute connected to a timsTOF Pro. The MS was programmed using a DDA-PASEF method with some similar instrument parameters to Thunder-DDA-PASEF (300 ms, 3 MS/MS frames) but without the high sensitivity mode and using a different isolation polygon. Importantly, the TIMS range was restricted to 0.6 – 1.3 $1/K_0$ (Fig. S1a). We downloaded the raw files and reprocessed them in PEAKS XPro. For simplicity, we show the combined results of the six files. Although 0.3% of all the peptides identified were singly charged, they were most likely false identifications. Indeed, singly charged peptides are always eluted at $> 1.3$ $1/K_0$ in all the analyses that we performed (e.g., see Fig. SS3). This highlights the importance of extending the TIMS analysis range up to 1.75 $1/K_0$ and including singly charged ion cloud in the isolation polygon.

We observed an unusually high proportion of multiply charged peptides ($\geq +3$) in this data set. To evaluate if those could contribute to the HLAI-ligandome, we evaluated the distributions of peptides when considering all the peptides, only the 8-13-mers or only the predicted binders S1a, b, c, respectively). The proportion of $\geq +3$ dropped from 24.7 % to 8.7% and 3.8%, indicating that those could be contaminant peptides. From the 9,430 peptides identified, only 52.2% of the peptides had the expected length size of 8-13 AAs S1d). Out of those 3,753 8-13-mers, only 48.7% were predicted binders S1e). These results cannot be directly compared to our results starting from 10-fold higher amounts of samples. However, they highlight the challenges of immunopeptidomics method optimization, from sample preparation to acquisition and data analysis.

# 3 Supplementary Figures

## 3.1 Fig. S1. Restricting the TIMS range results in the loss of singly charged peptides

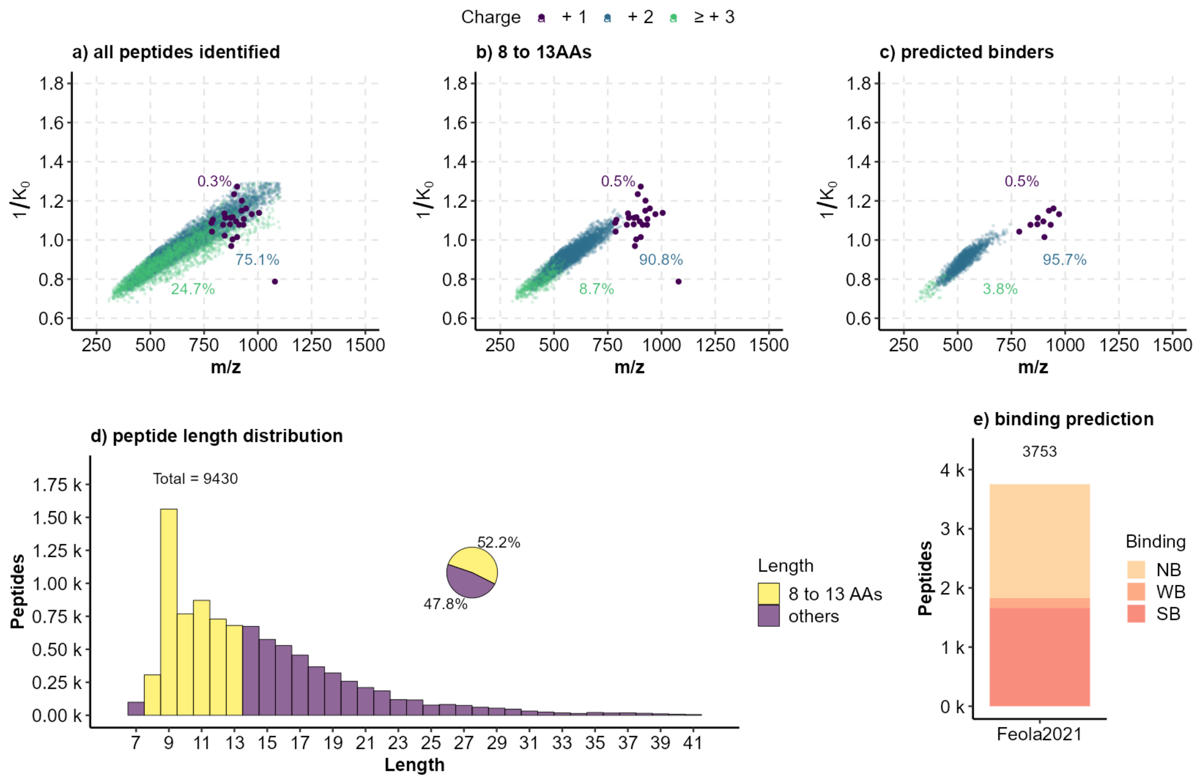


Figure S1: **HLA class I ligandome of JY cells prepared by a microfluidics system acquired in a timsTOF Pro MS but restricting the TIMS range to 0.6 – 1.3 1/$K_0$** (data from [2] was reprocessed in PEAKS XPro.) **(a, b, c):** peptides identified across the 1/$K_0$ vs m/z dimensions colored by charge state, including all peptides **(a)**, 8-13-mers **(b)**, or only predicted binders **(c)**. Singly charged peptides resulting likely from wrong identifications are higlighted with bigger and darker dots. **(d):** Length distribution and percentage of peptides (pie-charts) with 8 to 13 AAs or other lengths; cut-off at 41 AAs. **(e):** Number of 8-13-mer peptides identified in each workflow and the proportion predicted as strong-binders (SB, $rank \leq 0.5\%$), weak-binders (WB, $0.5\% < rank \leq 2\%$) or non-binders (NB, $rank > 2\%$) by NetMHCpan-4.1 [3].

## 3.2 Fig. S2. The HLAIp-tailored isolation polygon results in fewer long peptides (> 13 AAs)

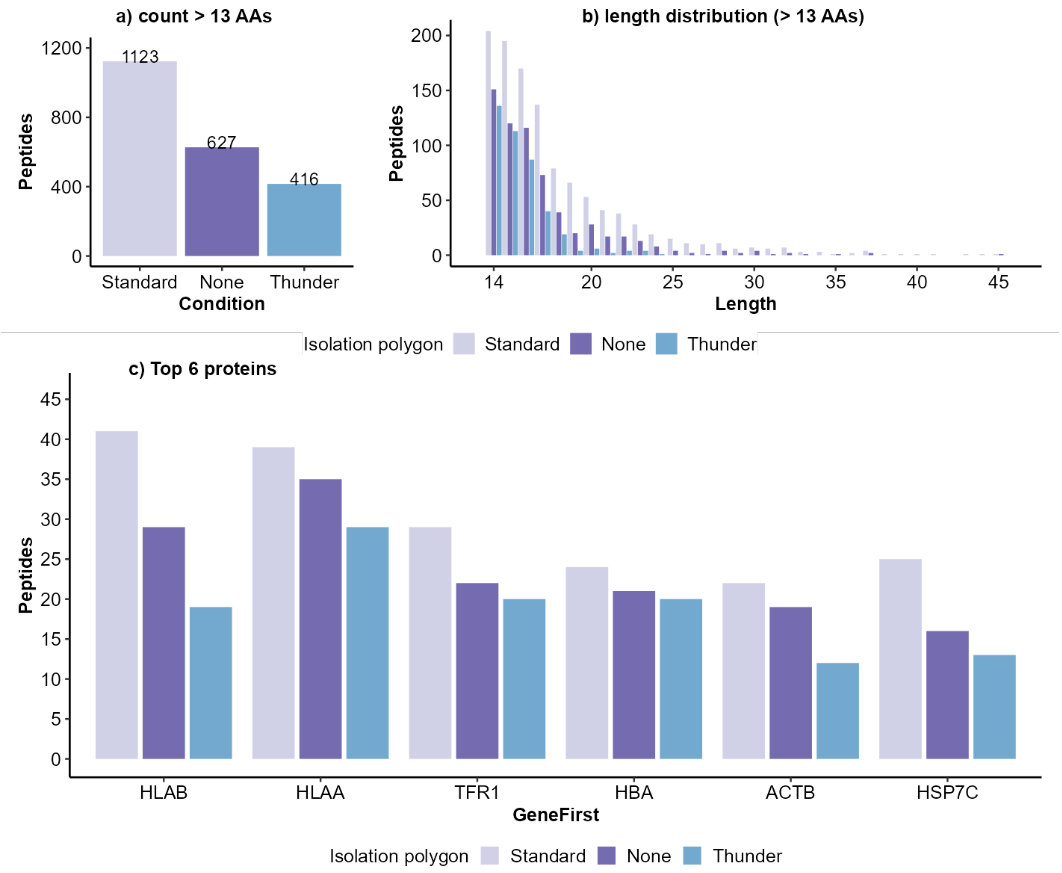


Figure S2: **Long multi-charged (z $= 2^+$ or $3^+$) peptides (> 13 AAs) in function of isolation polygon**. **a)** Total count of unique peptide sequences with > 13 AAs. **b)** Count in function of peptide length (AAs). Only 1 peptide with z $= 1^+$ and length > 13 AAs was identified, and it was detected only with None isolation polygon. **c)** Count of unique peptides (> 13 AAs) of the Top 6 proteins with the highest peptide count.

### 3.3 Fig. S3. Peptide distribution across the $1/K_0$ vs m/z dimensions for multiple samples acquired without or with the Thunder isolation polygon

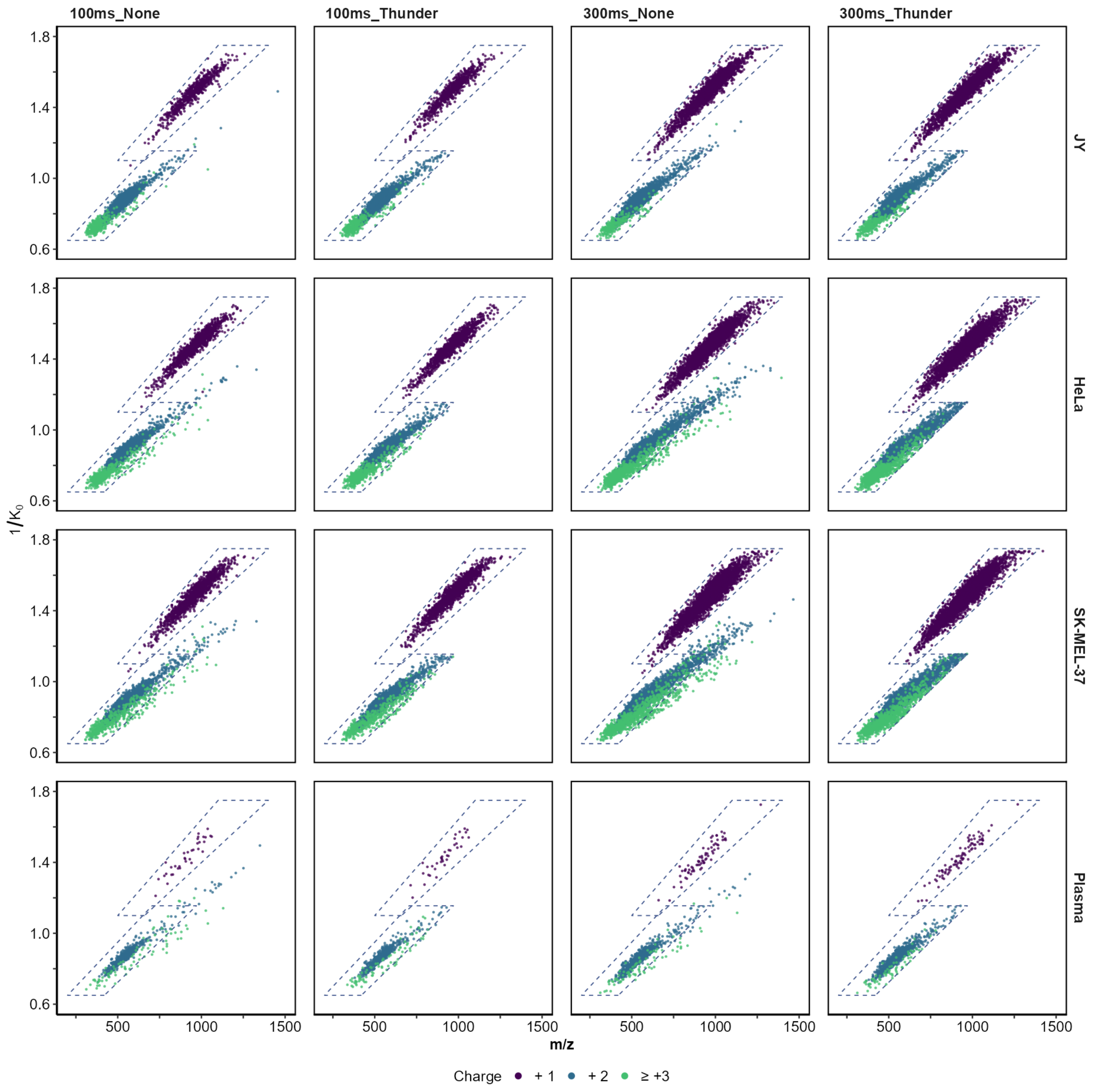


Figure S3: **Peptides identified across the $1/K_0$ vs m/z dimensions**. Each panel correspond to a sample type (columns) analyzed with a distinct DDA-PASEF method (rows). The methods correspond to the original (100ms TIMS) or optimized (300ms TIMS) DDA-PASEF settings, without (None) or with the Thunder isolation polygon. The dotted lines delimit the perimeter of the Thunder isolation polygon, although no polygon was used in the methods labeled as None.

## 3.4 Fig. S4. HLAIp distribution across the $1/K_0$ vs m/z dimensions for multiple samples acquired without or with the Thunder isolation polygon

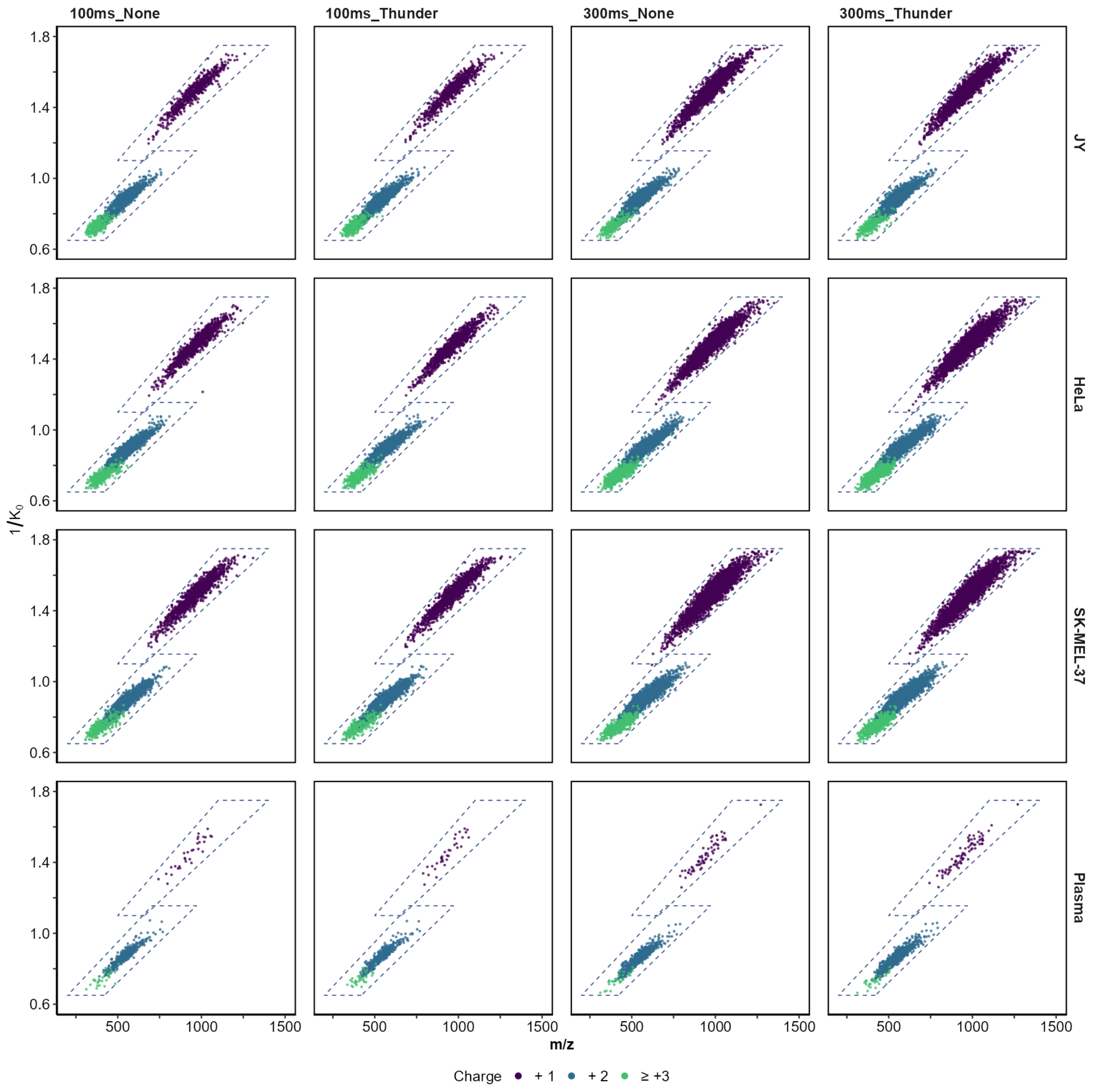


Figure S4: **Peptides predicted to bind the HLAI alleles of the respective samples (HLAIps), plotted across the $1/K_0$ vs m/z dimensions**. Each panel correspond to a sample type (columns) analyzed with a distinct DDA-PASEF method (rows). The methods correspond to the original (100ms TIMS) or optimized (300ms TIMS) DDA-PASEF settings, without (None) or with the Thunder isolation polygon. The dotted lines delimit the perimeter of the Thunder isolation polygon, although no polygon was used in the methods labeled as None.

### 3.5 Fig. S5. Dynamic range in 100 ms and 300 ms methods without and with the Thunder isolation polygon for multiple samples

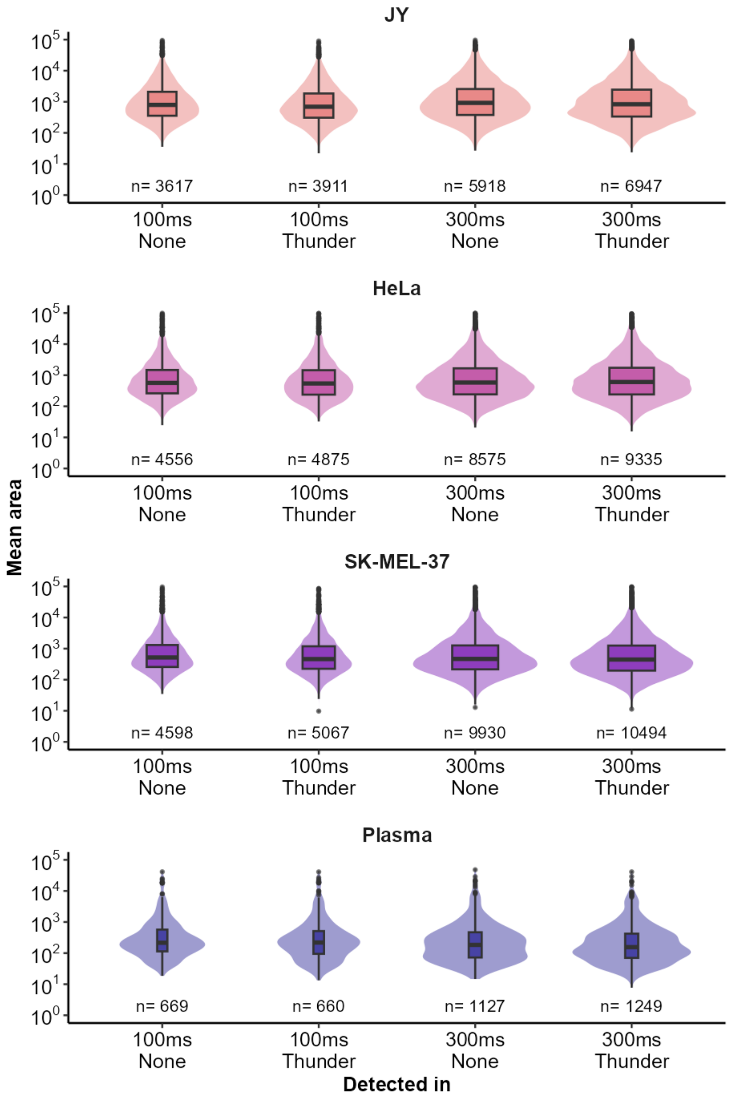


Figure S5: **Dynamic range**. Peptide area (mean of three replicates) distribution in function of rescoring result represented as violin and boxplots (center line, median; box limits, upper and lower quartiles; whiskers, 1.5x interquartile range). Values represent the total number of peptides (with modifications) identified.

### 3.6 Fig. S6. HLAIp sequence motifs of the 9-mers dentified in JY, HeLa, SK-MEL-37, and plasma

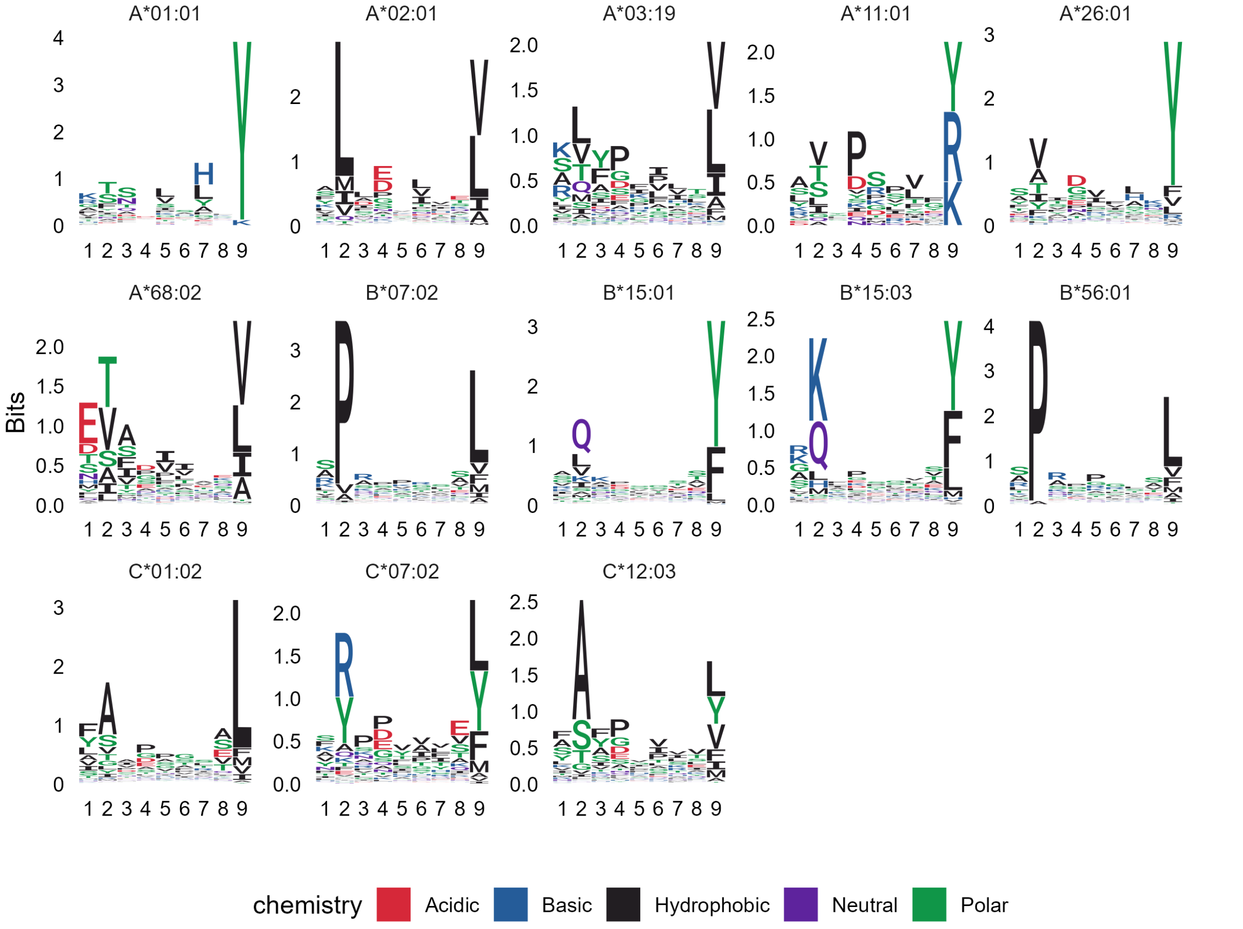


Figure S6: **Peptide sequence motifs of the 9-mers predicted to bind the respective HLA alleles**. Each panel correspond to all the predicted binders identified across JY, HeLa, SK-MEL-37, and plasma samples with the four different methods (100ms_None, 100ms_Thunder, 300ms_None and 300ms_Thunder). Sequence logos were generated using ggseqlogo [4]

## 3.7 Fig. S7. HLA-specific HLAIp distribution across the $1/K_0$ vs m/z dimensions for multiple samples acquired without or with the Thunder isolation polygon

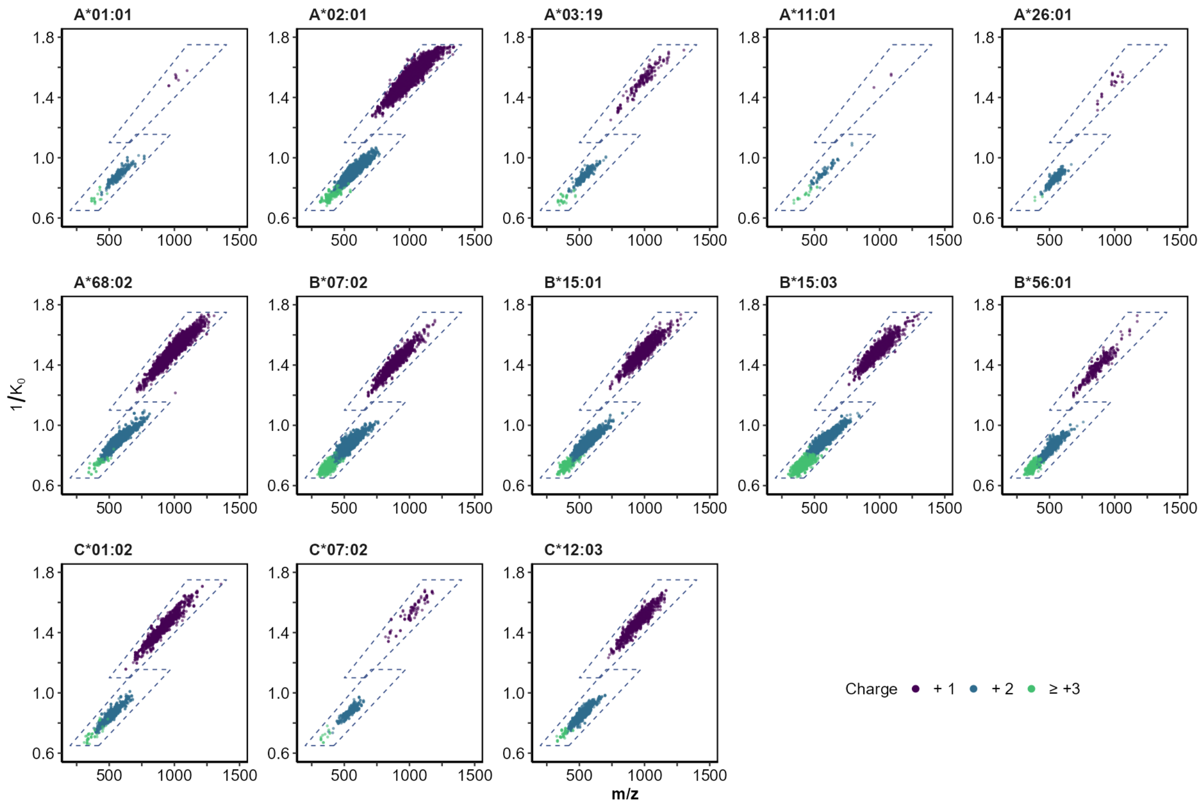


Figure S7: **Peptides predicted to bind the HLAI alleles of the respective alleles, plotted across the $1/K_0$ vs m/z dimensions**. Each panel correspond to all the predicted binders identified across JY, HeLa, SK-MEL-37, and plasma samples with the four different methods (100ms_None, 100ms_Thunder, 300ms_None and 300ms_Thunder). The dotted lines delimit the perimeter of the Thunder isolation polygon.

## 3.8 Fig. S8. Charge distribution in function of the HLA allotype

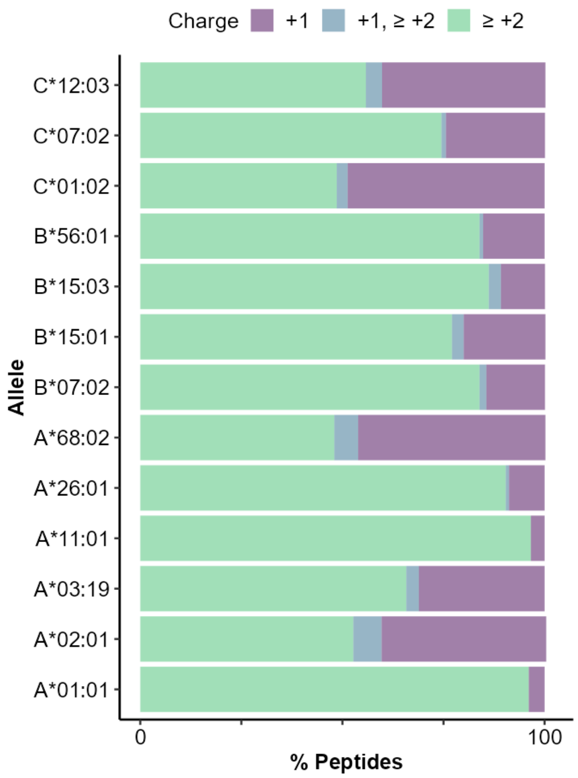


Figure S8: **Charge distribution in function of the HLA allele**. Charge state distribution of the HLAIps (peptides considering modifications) in function of their predicted HLA allele binding. Each bar correspond to all the predicted binders identified across JY, HeLa, SK-MEL-37, and plasma samples with the four different methods (100ms_None, 100ms_Thunder, 300ms_None and 300ms_Thunder).

## 3.9 Fig. S9. Evaluation of the timsTOF prediction models

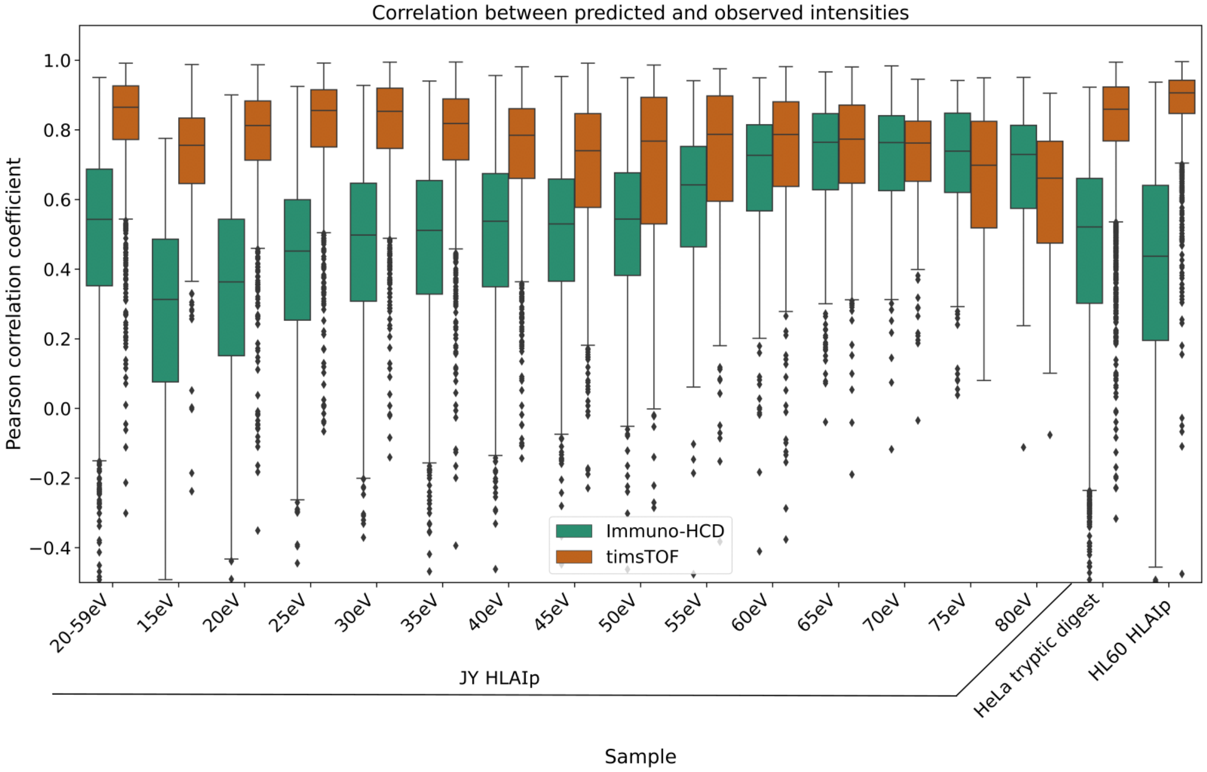


Figure S9: **Evaluation of the timsTOF prediction models compared to the HCD immunopeptidomics model** using the Pearson correlation coefficient (PCC) between predicted and observed intensities on data from JY HLAIp samples acquired with the standard collision energy (CE) ramp ($20 - 59eV, 0.65 - 1.60Vs/cm^2$), or a series of fixed CEs from 15 to 80 eV (acquired in Tenzer lab), as well as an independent analysis of a HeLa tryptic digest and an HL60 HLAIp sample acquired as mentioned in the Methods (acquired in Carapito lab).

## 3.10 Fig. S10. Peptide and protein overlap between biological replicates of JY and Raji

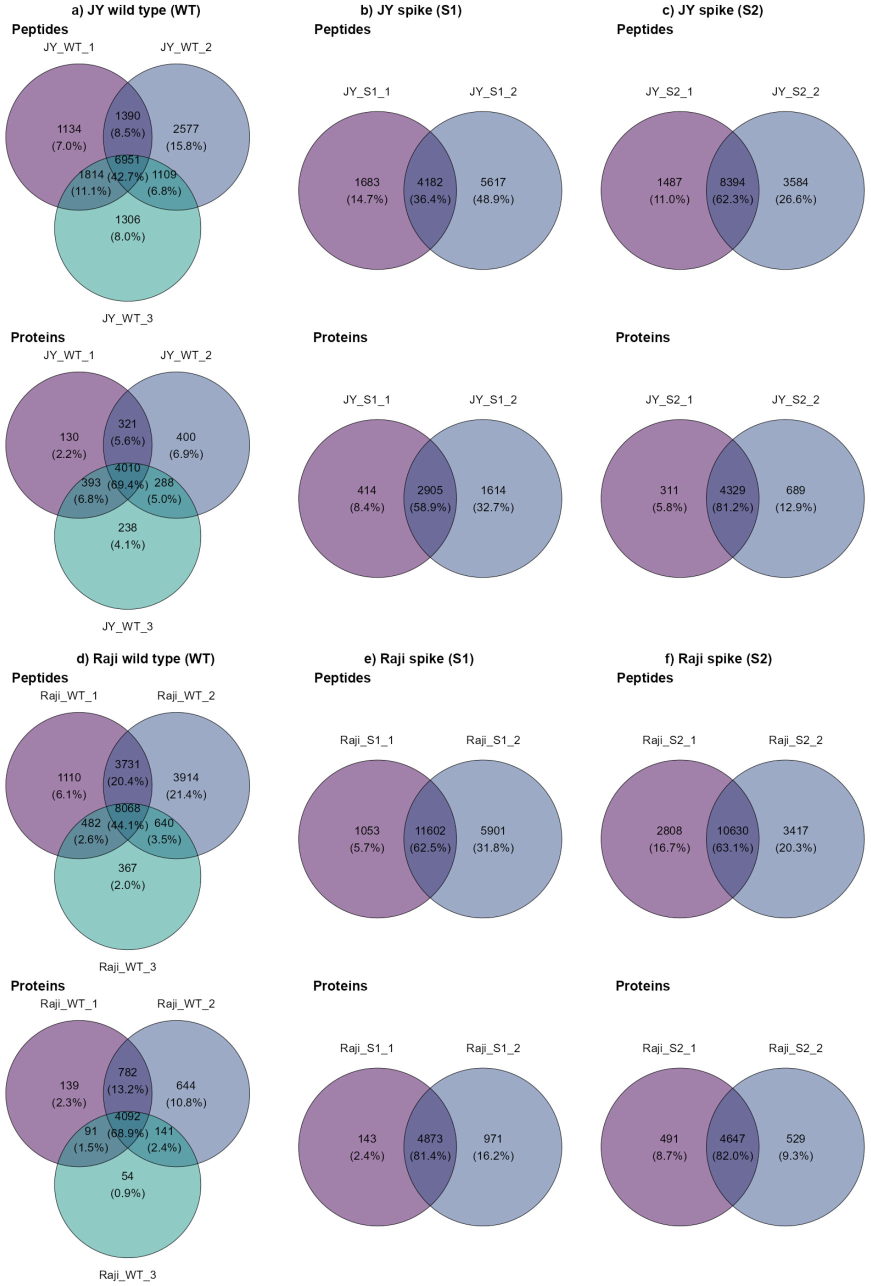


Figure S10: **8 to 13-mer peptide and corresponding protein overlap between biological replicates of JY (a, b, c) and Raji (d, e, f).** Venn plots showing the overlap of peptides identified in each biological replicate of JY and Raji for each genotype. WT = wild type, S1 = transfected with SARS-CoV-2 spike segment one, or S2 = spike segment 2.

## 3.11 Fig. S11. Peptide characteristics of HLAIps in the JY and Raji spike-transfected experiment by allotype

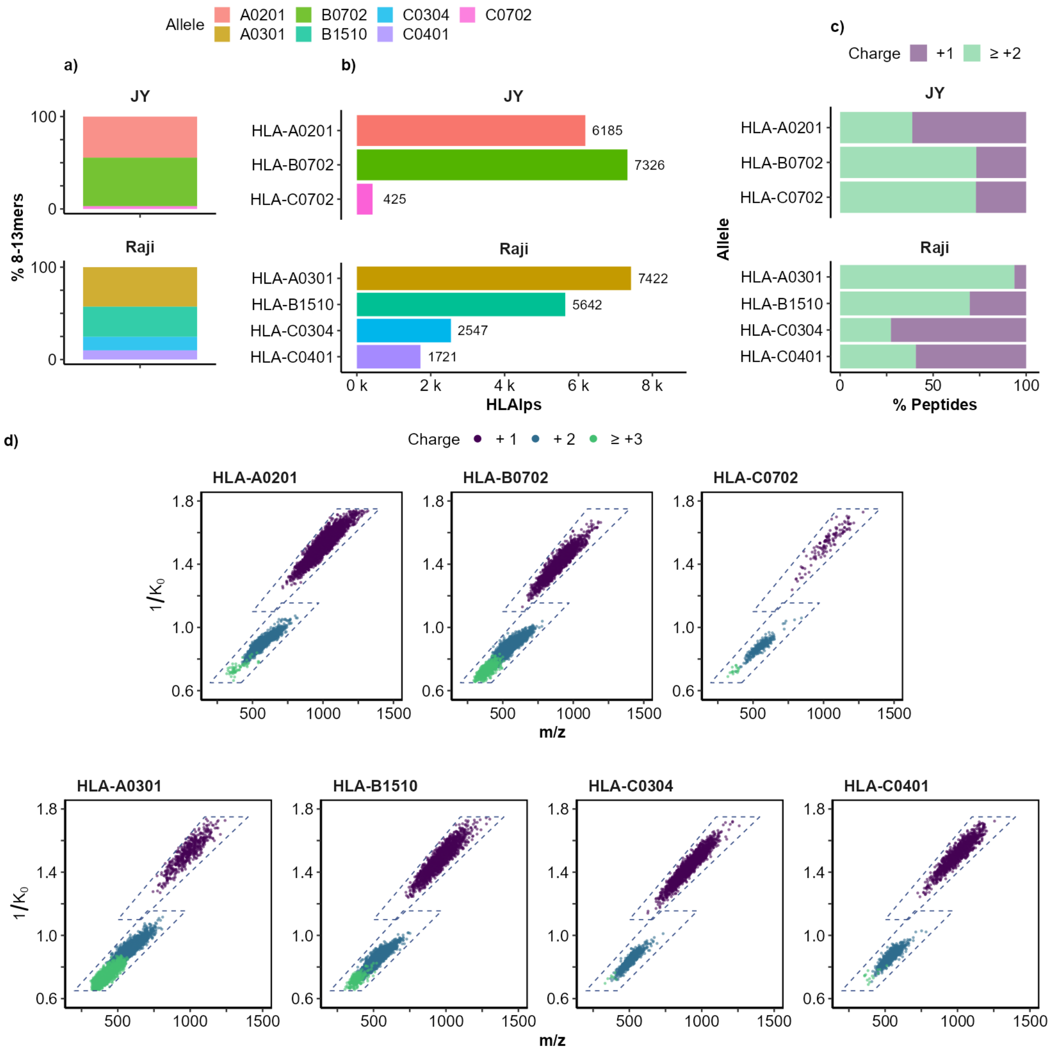


Figure S11: **Peptide characteristics of HLAIps in the JY and Raji spike-transfected experiment by allotype (a)** Proportion of 8-13-mers predicted to bind each of the HLA alleles of each cell line. **(b)** Total HLAIps detected in each cell line. **(c)** Charge state distribution of the HLAIps (peptides considering modifications) in function of their predicted HLA allele binding. **(d)** Peptides predicted to bind the HLAI alleles of the respective alleles, plotted across the $1/K_0$ vs m/z dimensions; the dotted lines delimit the perimeter of the Thunder isolation polygon.

## 3.12 Fig. S12. Hierarchical structure visualization of the GO enrichment analysis of JY Raji common proteins covered by HLA class I ligands

Hierarchical structure visualization of the (GO) enrichment analysis using GOrilla [5] for the proteins identified in the immunopeptidome (HLA class I ligands) of JY and Raji. The plots shown in the following pages include the results for the three different ontologies, in this order: function (a), component (b), and process (c).

a)

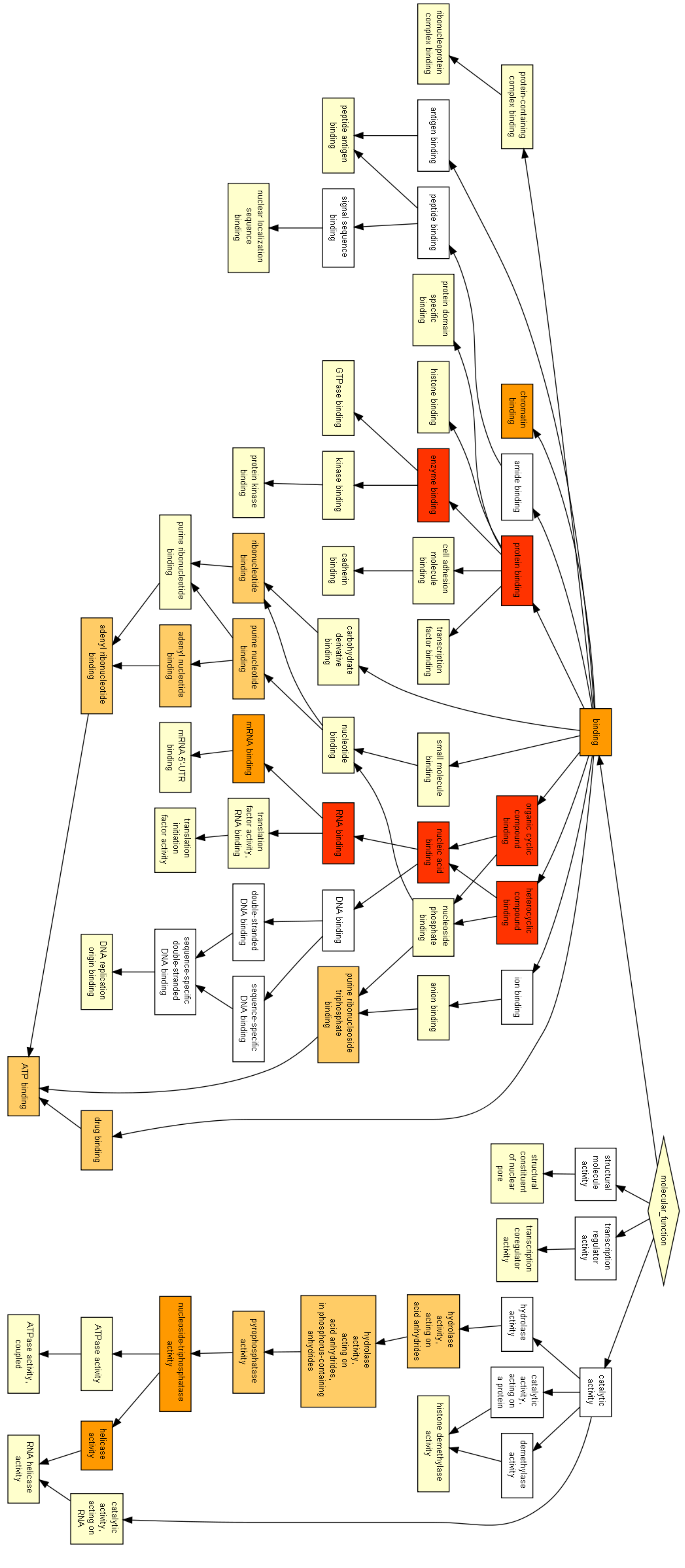

molecular_function
binding
ribonucleoprotein complex binding
protein-containing complex binding
antigen binding
peptide antigen binding
peptide binding
signal sequence binding
nuclear localization sequence binding
protein domain specific binding
chromatin binding
histone binding
GTPase binding
enzyme binding
amide binding
kinase binding
protein kinase binding
protein binding
cell adhesion molecule binding
cadherin binding
transcription factor binding
carbohydrate derivative binding
ribonucleotide binding
purine ribonucleotide binding
purine nucleotide binding
adenyl nucleotide binding
adenyl ribonucleotide binding
nucleotide binding
small molecule binding
mRNA binding
mRNA 5'-UTR binding
organic cyclic compound binding
nucleic acid binding
RNA binding
translation factor activity, RNA binding
translation initiation factor activity
heterocyclic compound binding
nucleoside phosphate binding
DNA binding
double-stranded DNA binding
sequence-specific double-stranded DNA binding
DNA replication origin binding
sequence-specific DNA binding
ion binding
anion binding
purine ribonucleoside triphosphate binding
ATP binding
drug binding
structural molecule activity
structural constituent of nuclear pore
transcription regulator activity
transcription coregulator activity
catalytic activity
hydrolase activity
hydrolase activity, acting on acid anhydrides
hydrolase activity, acting on acid anhydrides, in phosphorus-containing anhydrides
pyrophosphatase activity
nucleoside-triphosphatase activity
ATPase activity
ATPase activity, coupled
helicase activity
RNA helicase activity
catalytic activity, acting on RNA
catalytic activity, acting on a protein
demethylase activity
histone demethylase activity

b)

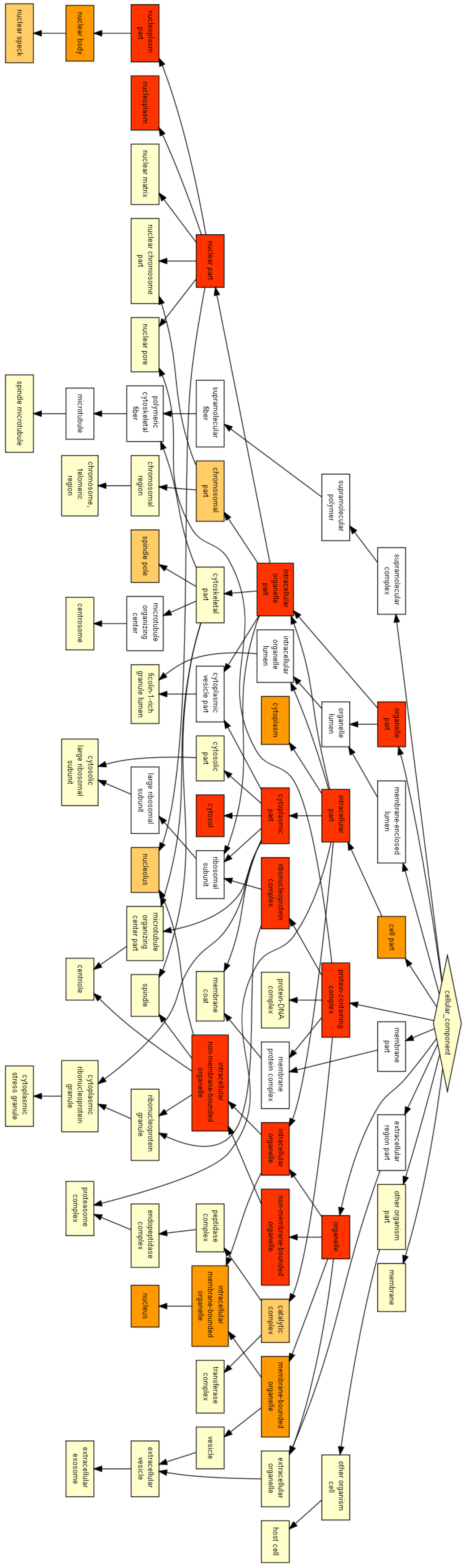

nuclear speck
nuclear body
nucleoplasm part
nucleoplasm
nuclear matrix
nuclear chromosome part
nuclear part
nuclear pore
spindle microtubule
microtubule
polymeric cytoskeletal fiber
supramolecular fiber
chromosome, telomeric region
chromosomal region
chromosomal part
supramolecular polymer
spindle pole
cytoskeletal part
intracellular organelle part
supramolecular complex
centrosome
microtubule organizing center
intracellular organelle lumen
ficolin-1-rich granule lumen
cytoplasmic vesicle part
cytoplasm
organelle lumen
organelle part
cytosolic large ribosomal subunit
cytosolic part
large ribosomal subunit
cytosol
cytoplasmic part
intracellular part
membrane-enclosed lumen
nucleolus
ribosomal subunit
ribonucleoprotein complex
microtubule organizing center part
cell part
centriole
spindle
membrane coat
protein-DNA complex
protein-containing complex
cellular_component
membrane part
membrane protein complex
cytoplasmic stress granule
cytoplasmic ribonucleoprotein granule
intracellular non-membrane-bounded organelle
ribonucleoprotein granule
intracellular organelle
extracellular region part
proteasome complex
endopeptidase complex
peptidase complex
non-membrane-bounded organelle
organelle
other organism part
membrane
nucleus
intracellular membrane-bounded organelle
catalytic complex
transferase complex
membrane-bounded organelle
vesicle
extracellular vesicle
extracellular exosome
extracellular organelle
other organism cell
host cell

c)

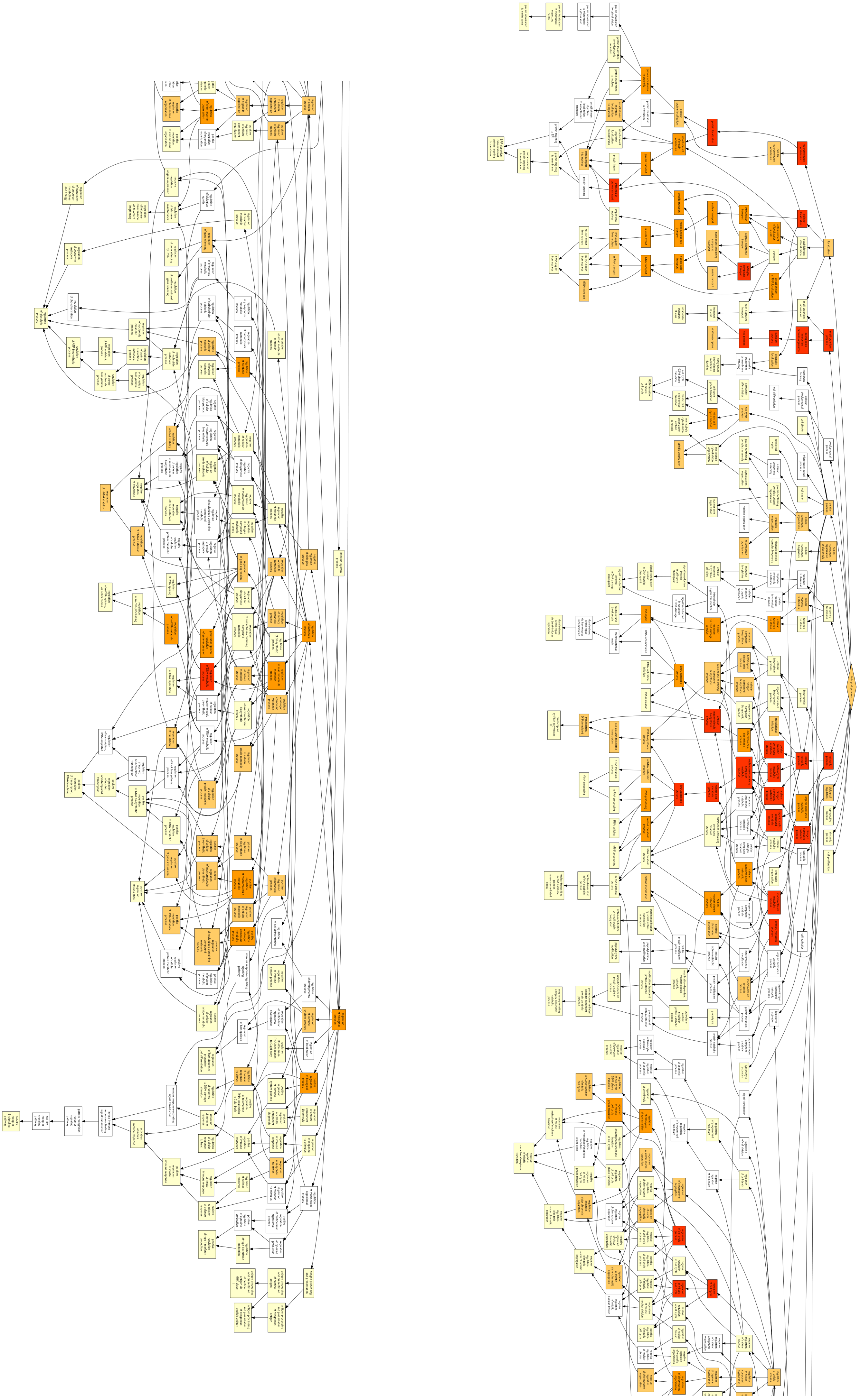

### 3.13 Fig. S13. Spike immunopeptides fragmentation spectra comparison vs. synthetic or predicted

The figures in the following pages show the mirrored fragmentation spectra (generated with USE [6]) showing the spectra from endogenous peptides at the top and synthetic or predicted spectra for HLA class I ligands of SARS-CoV-2 spike detected in JY or Raji cells transfect to express the protein. PCC = Pearson's correlation coefficient, SA = spectral (contrast) angle.

AIHVSGTNGTK_+2

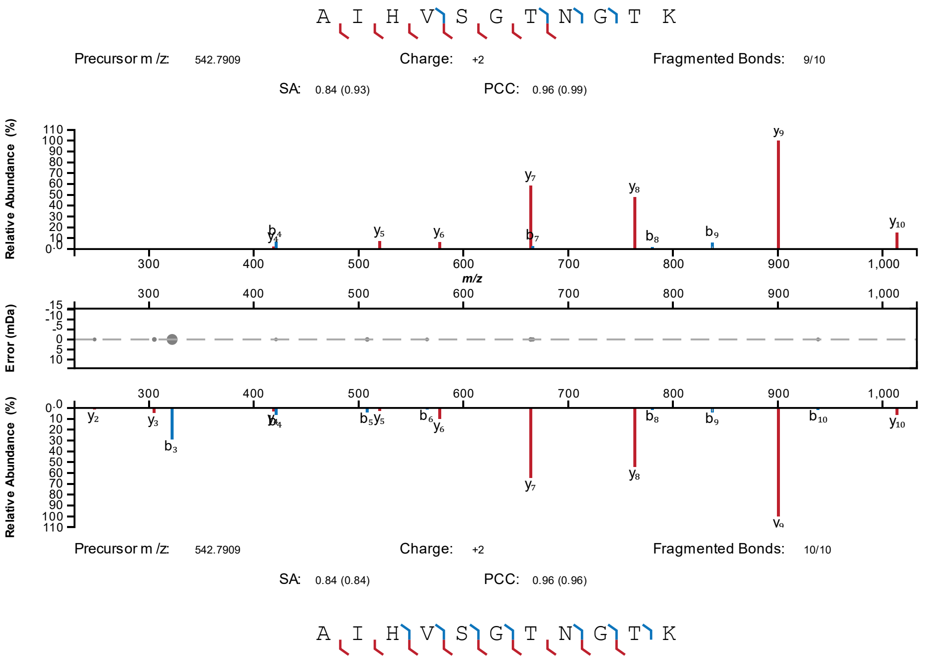


AIHVSGTNGTK_+3

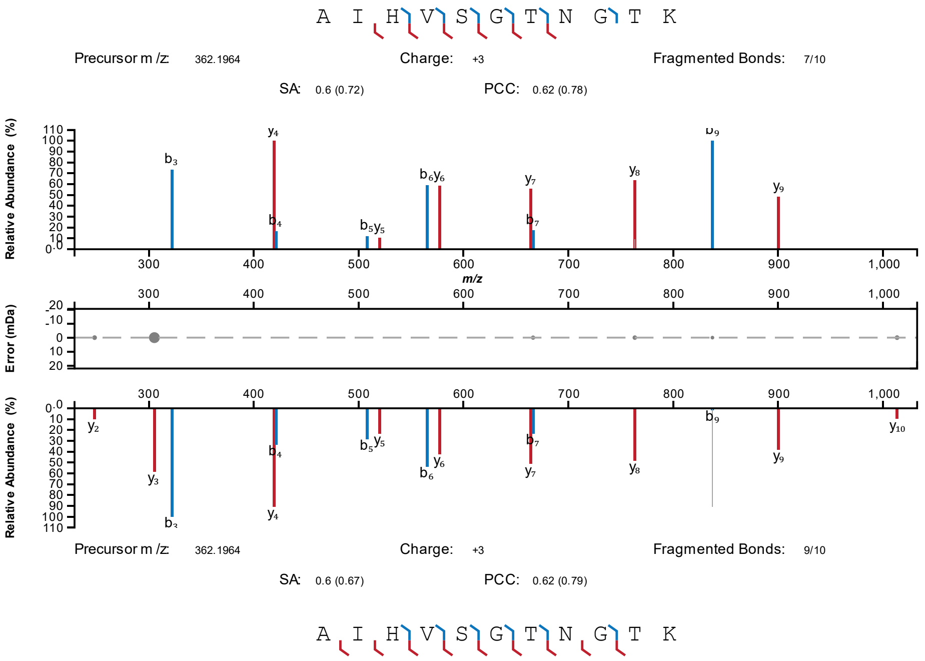

APHGVVFL_+1

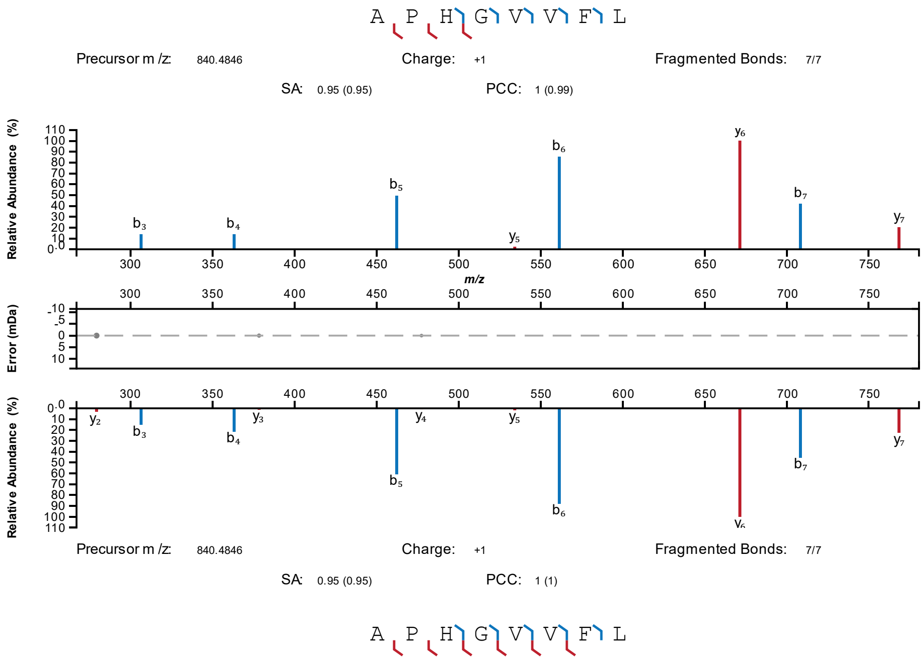


GVLTESNKK_+2

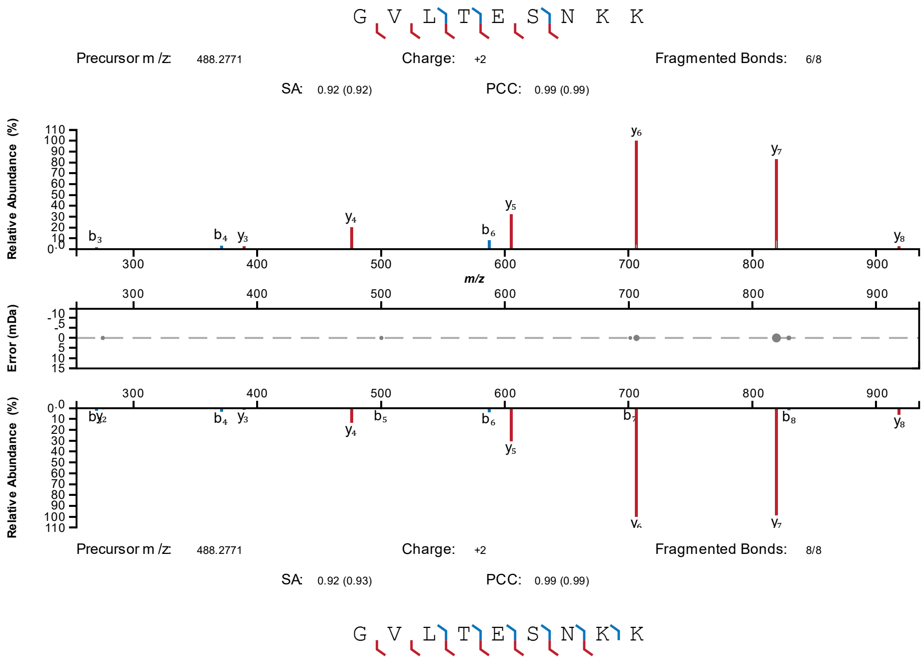

GVYFASTEK_+1

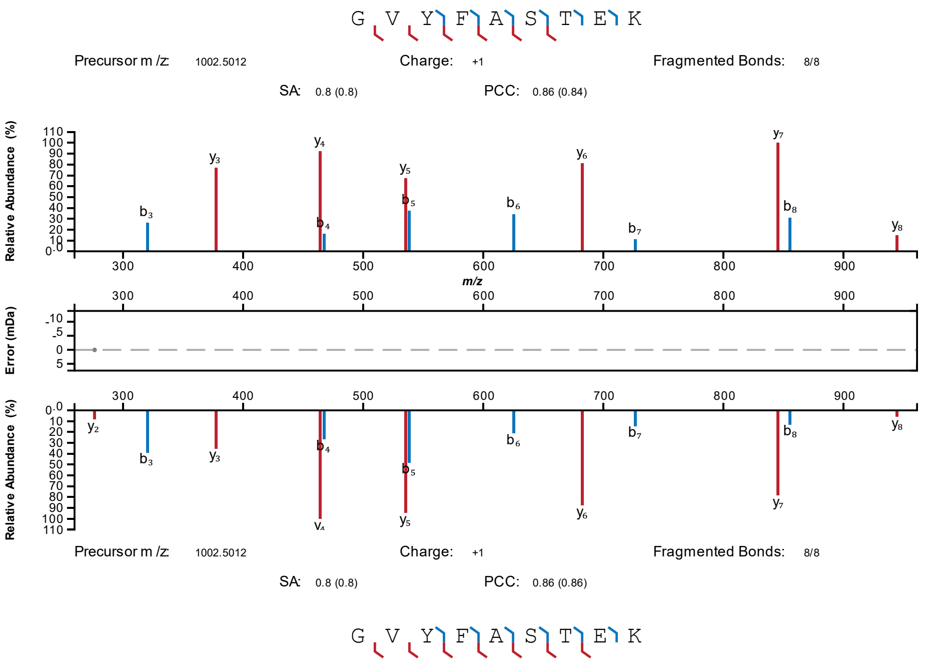


GVYFASTEK_+2

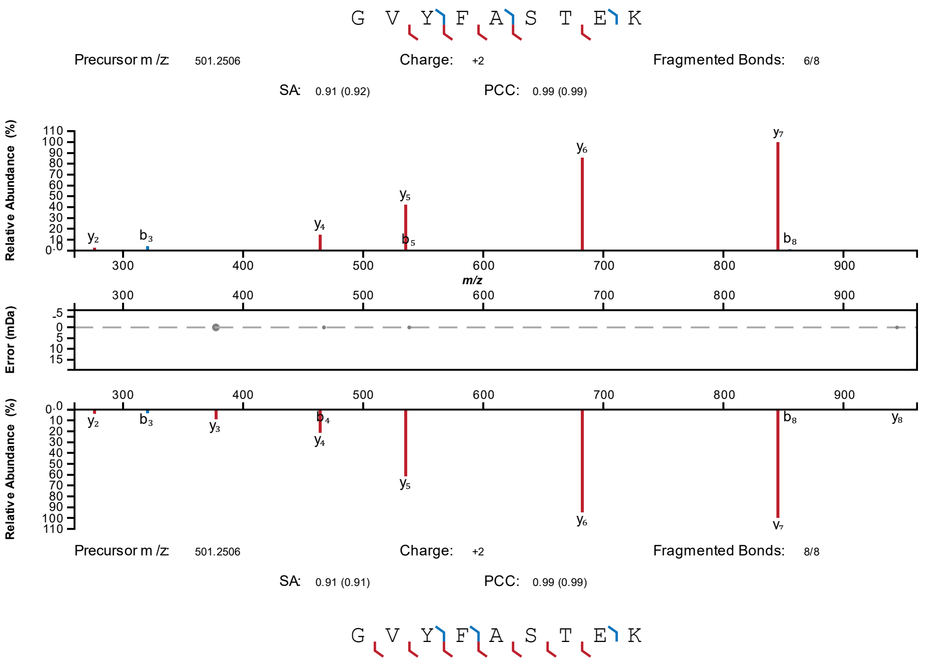

IPTNFTISV_+1

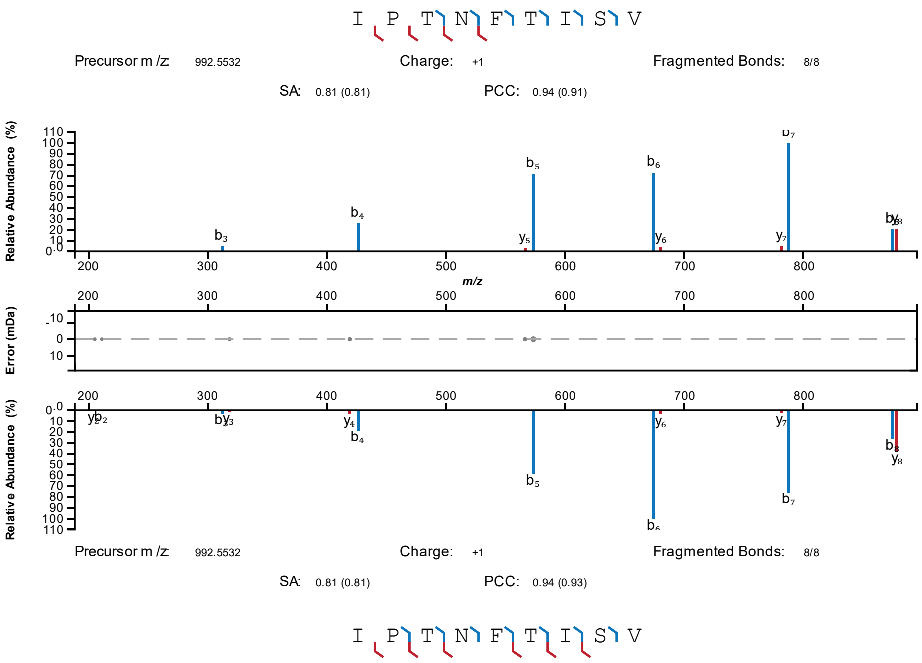


NRALTGIAV_+2

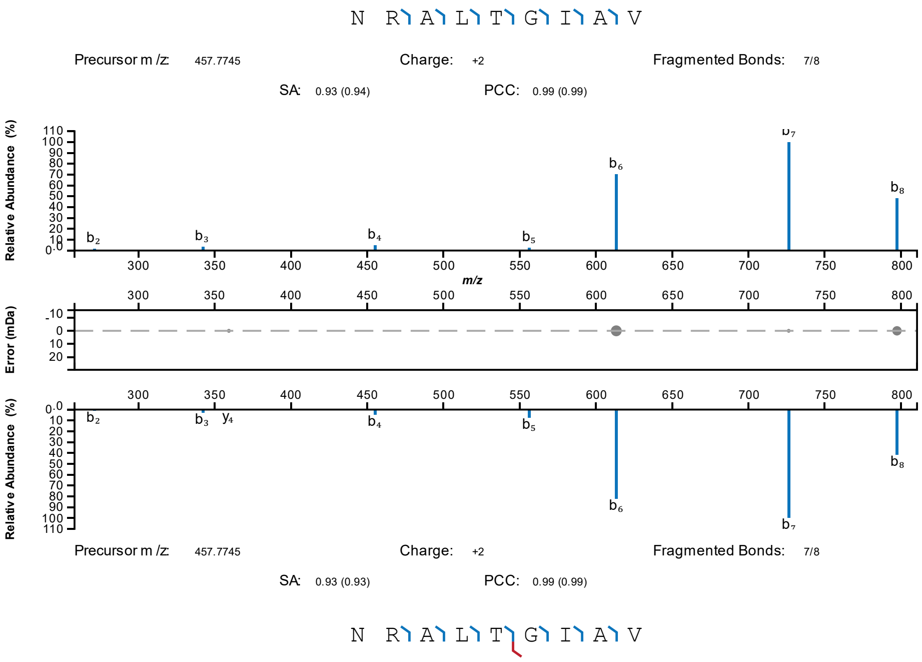

# RLDKVEAEV_+2

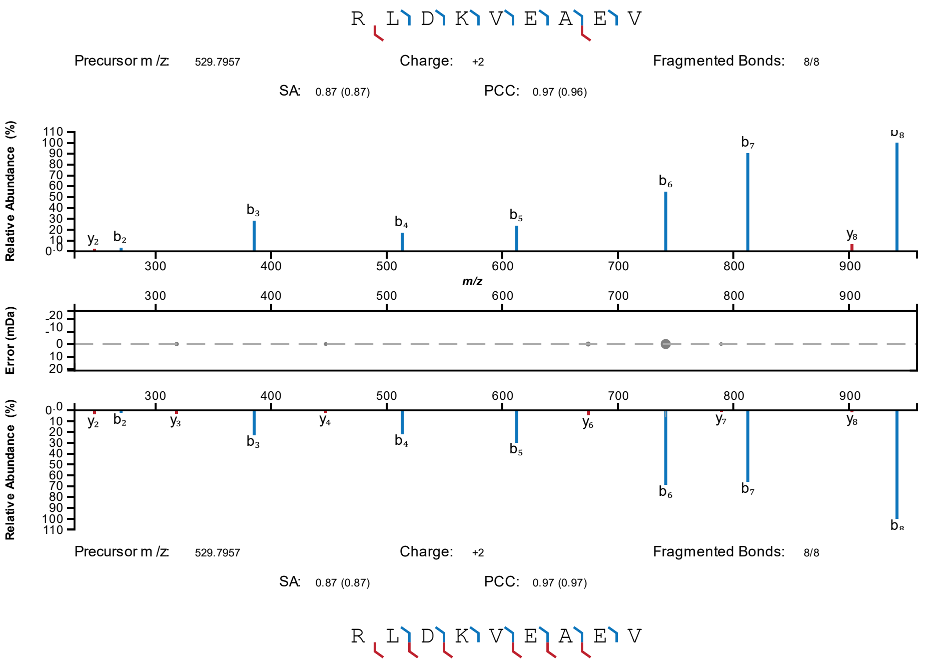


# RLQSLQTYV_+2

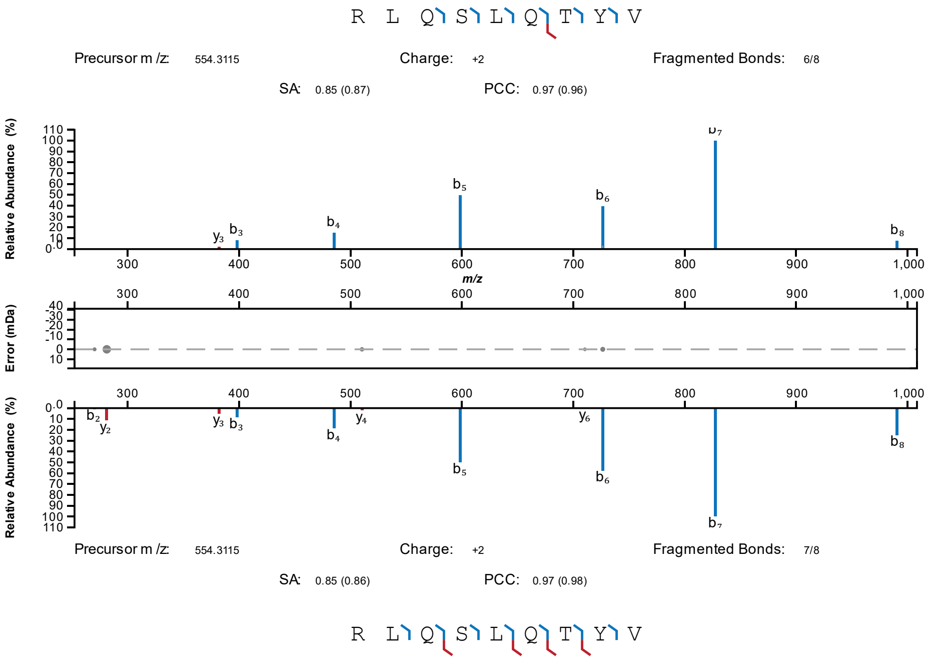

RQIAPGQTGK_+2

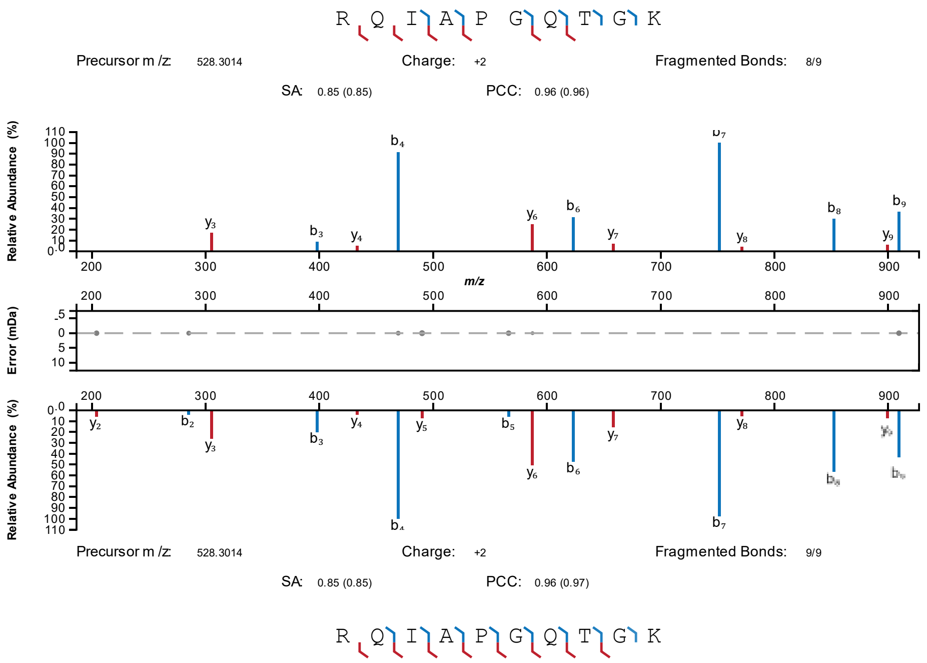


RVYSTGSNVFQTR_+2

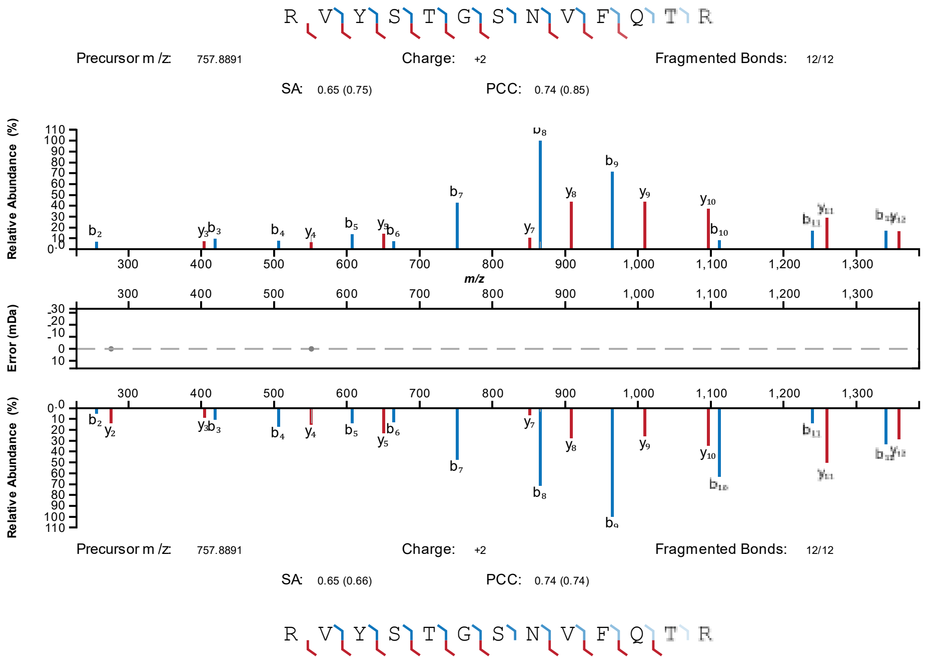

RVYSTGSNVFQTR_+3

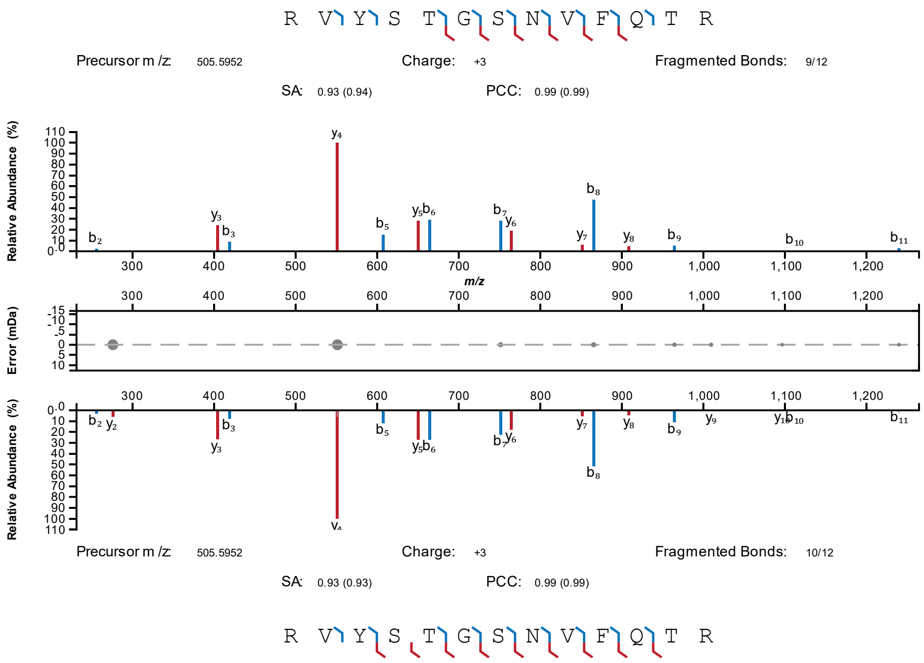


SIIAYTM(UniMod35)SL_+1_JY

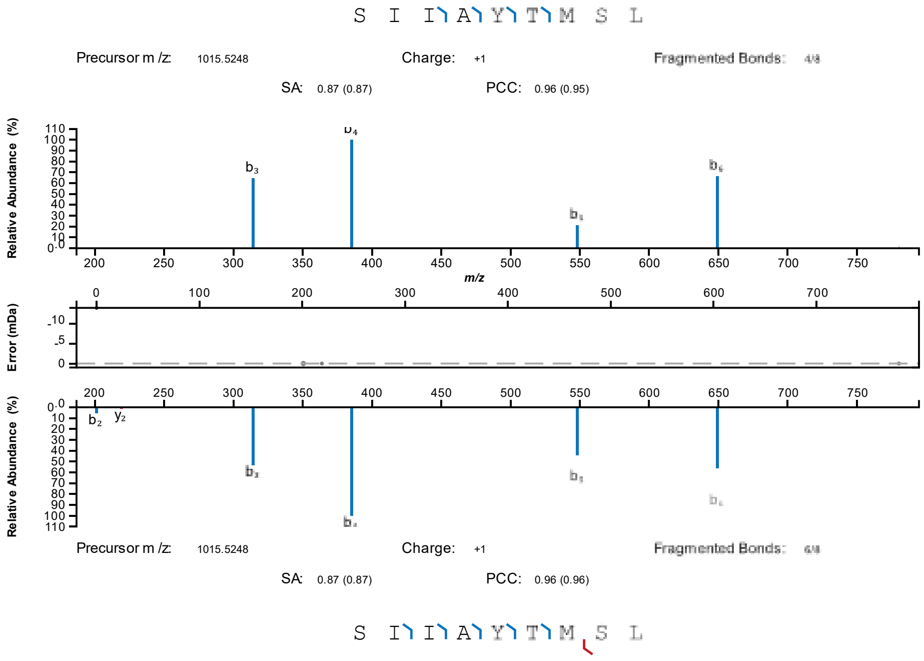

## SIIAYTM(UniMod35)SL_+1_Raji

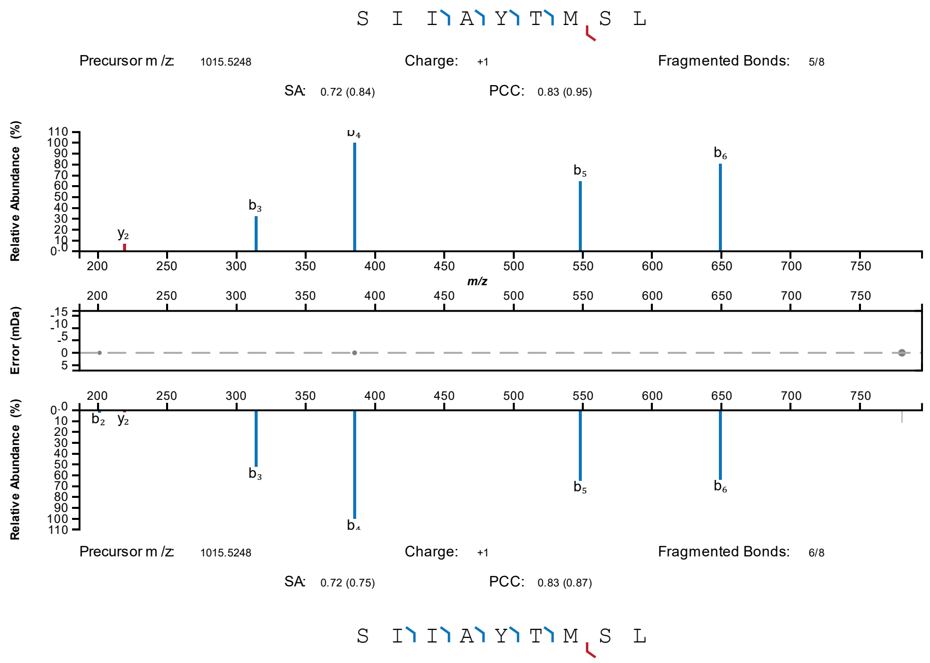


## SIIAYTMSL_+1_JY

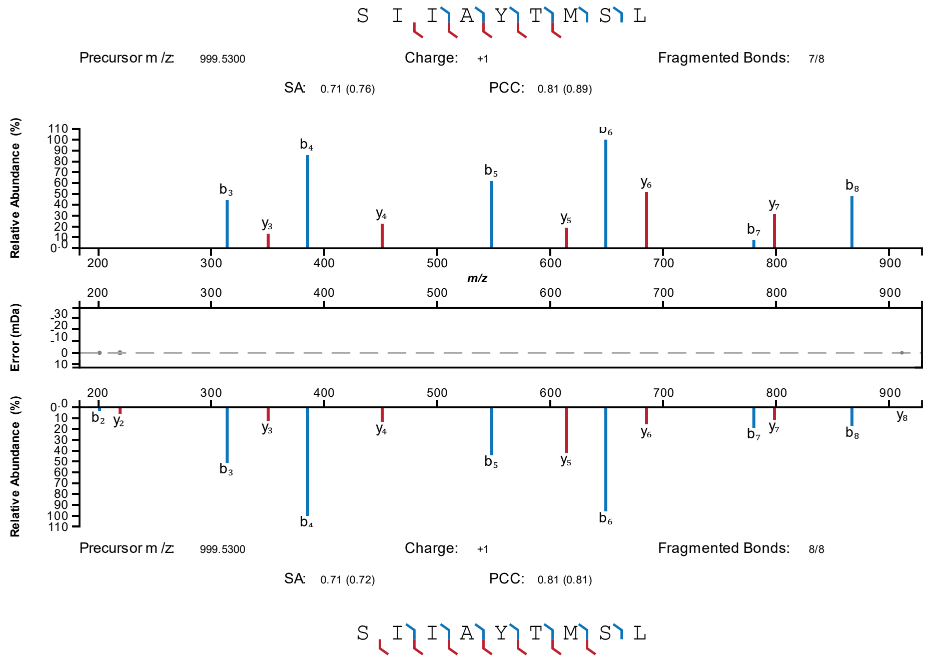

## SIIAYTMSL_+1_Raji

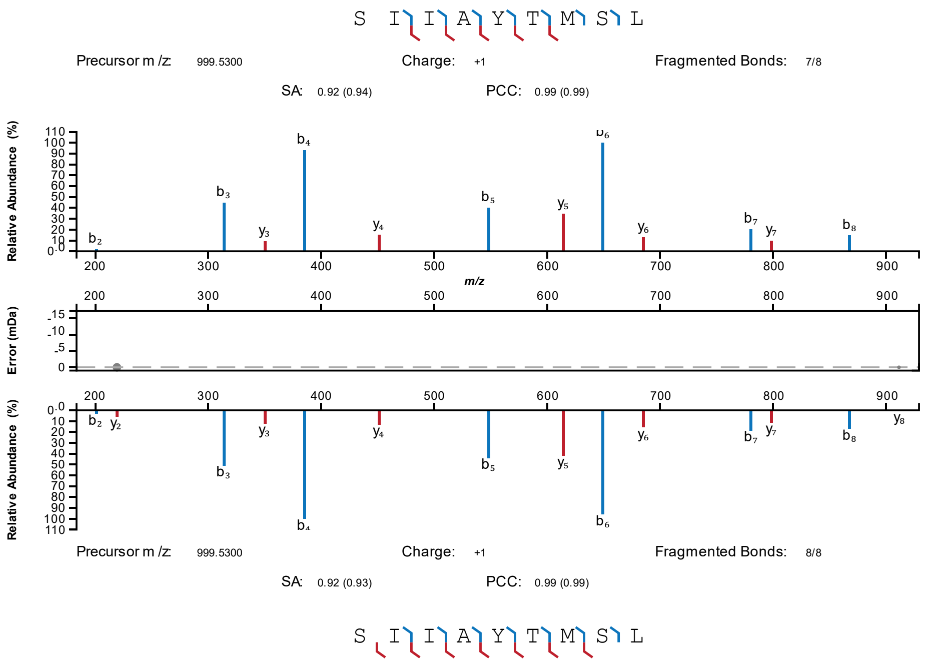


## TLKSFTVEK_+2

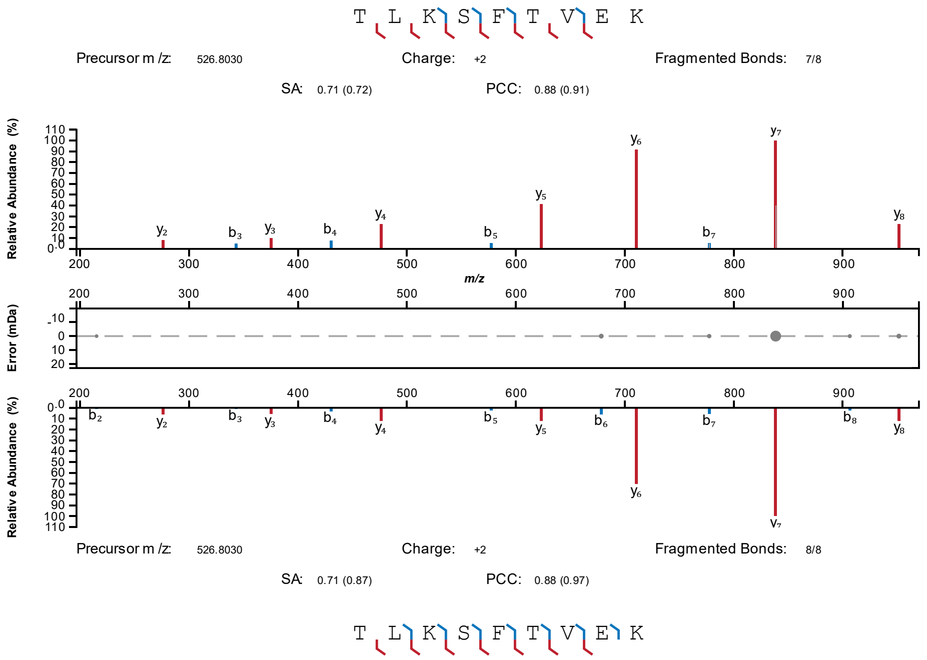

TLKSFTVEK_+3

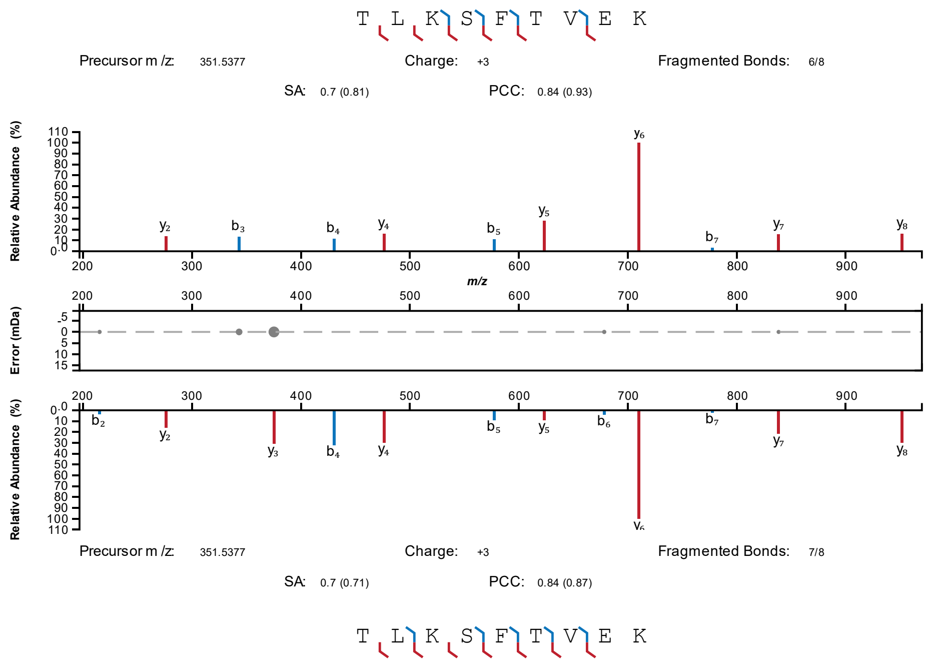


VLYNSASFSTFK_+2

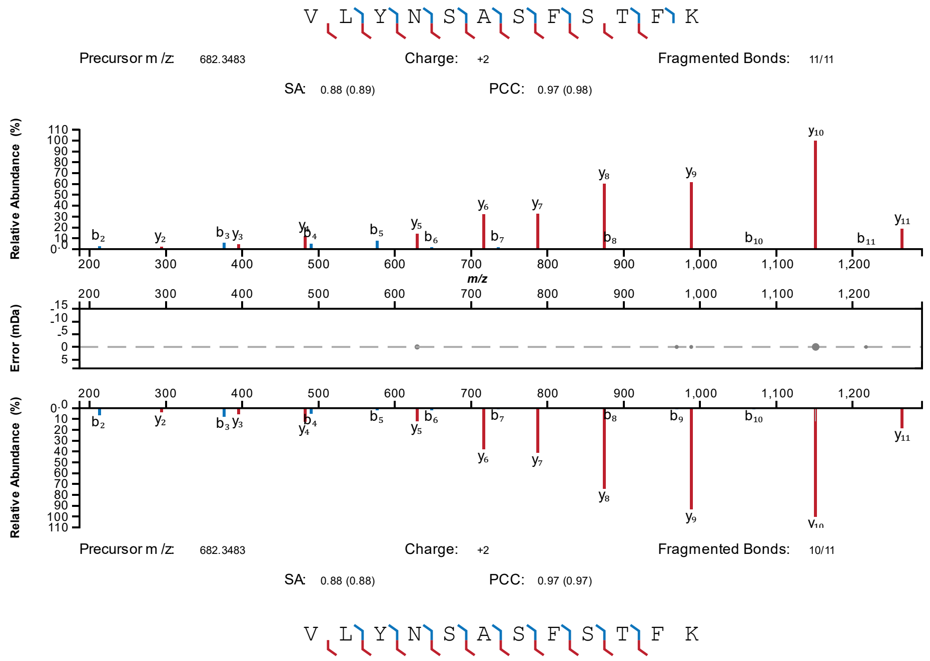

VTYVPAQEK_+1

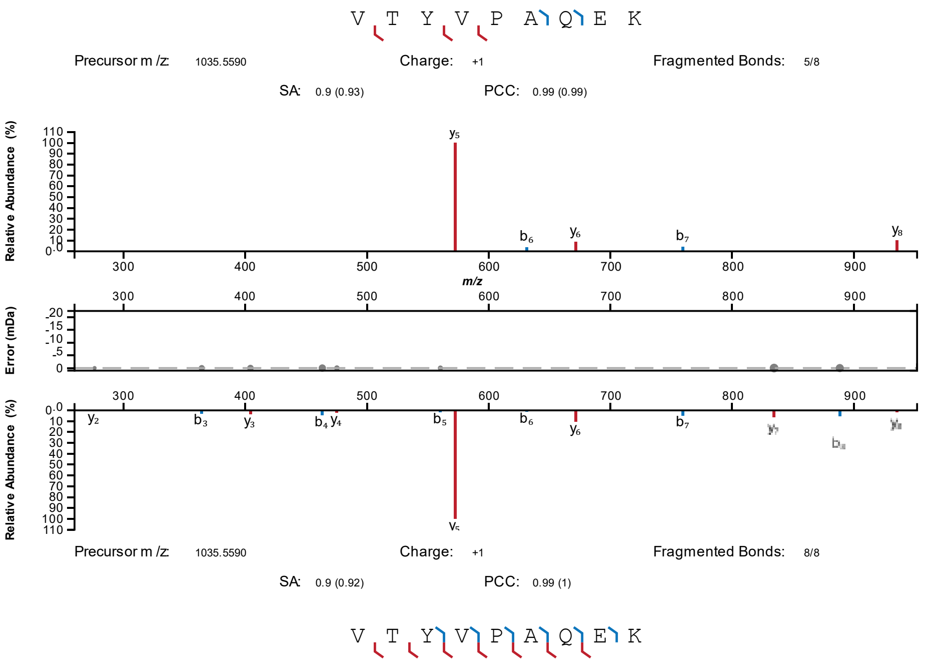


VTYVPAQEK_+2

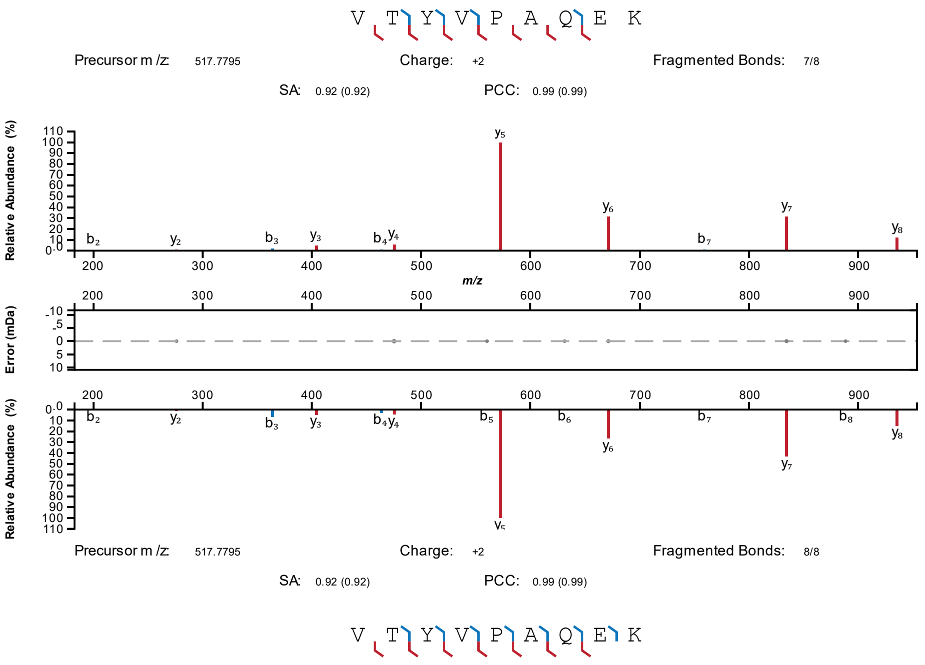

VYDPLQPEL_+1

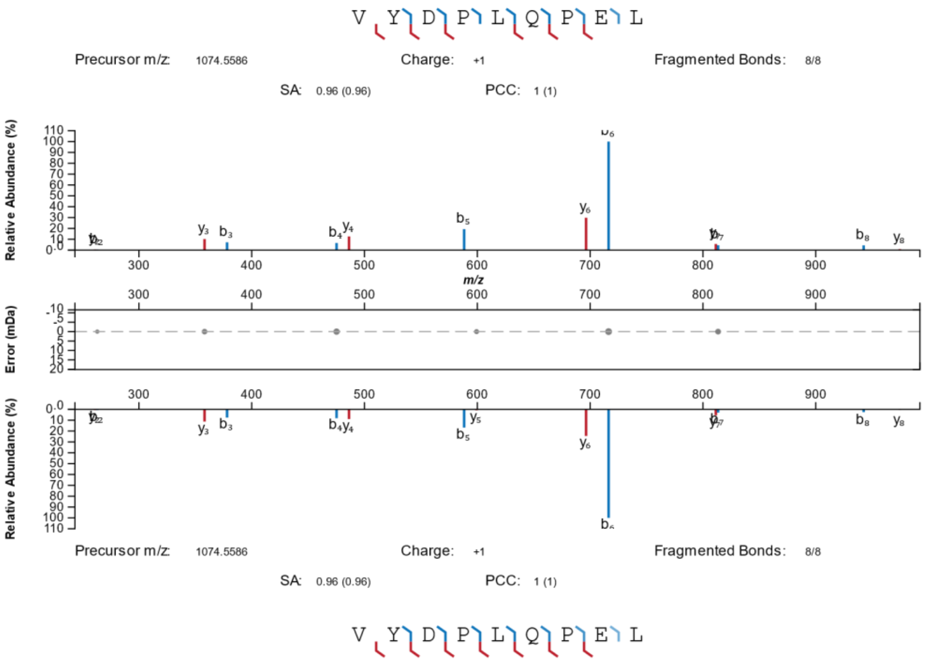


VYDPLQPEL_+2

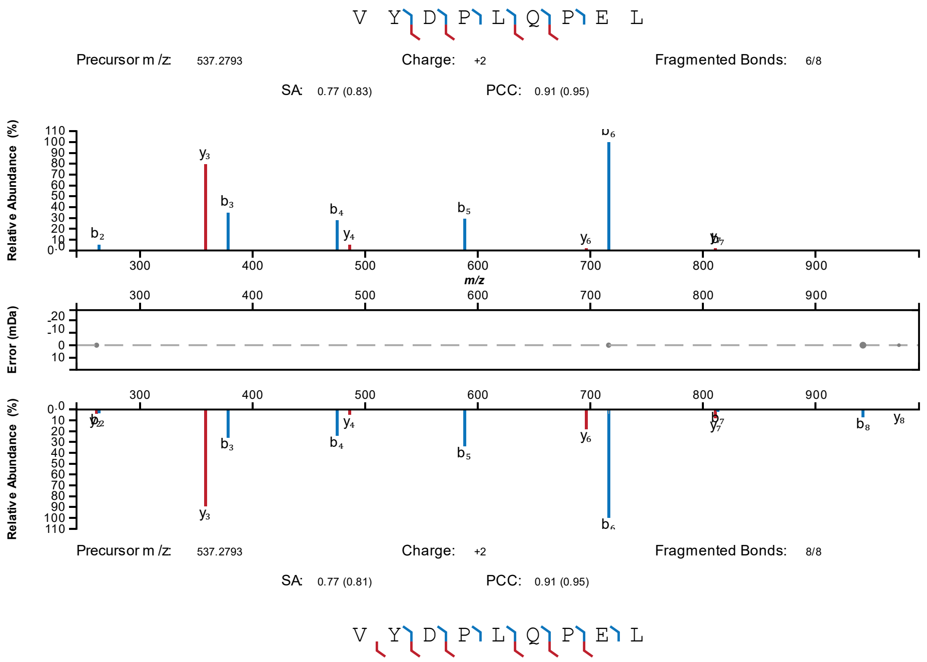

YGVSPTKL_+1

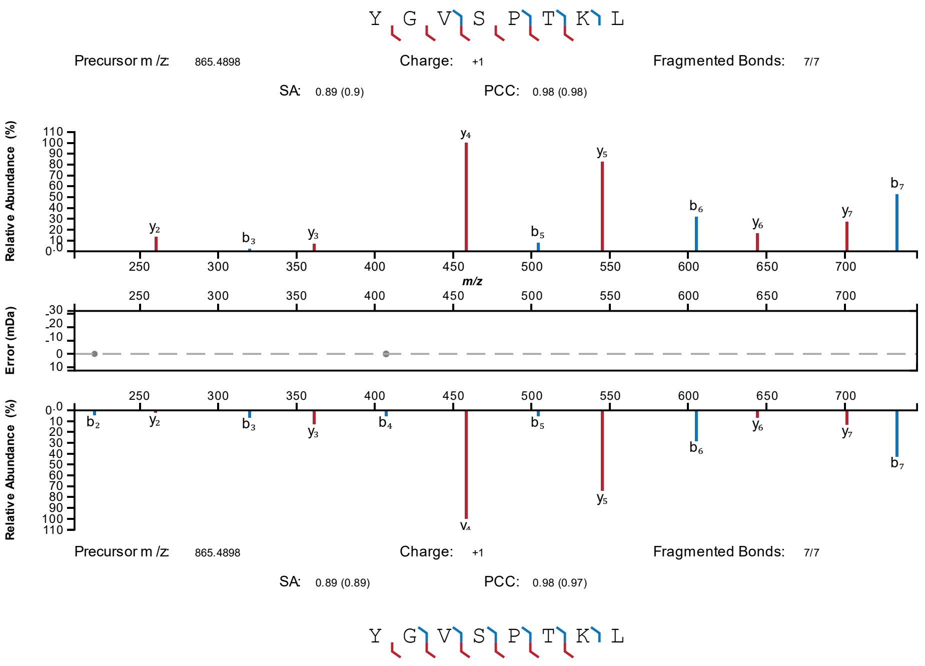


YGVSPTKL_+2

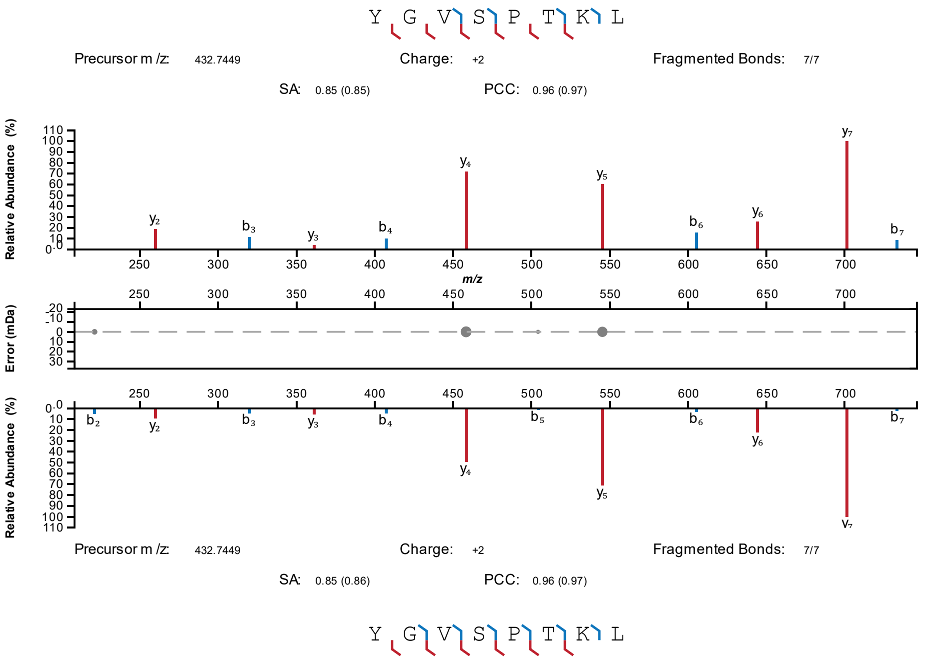

# References


[1] J. Shang, G. Ye, K. Shi, Y. Wan, C. Luo, H. Aihara, Q. Geng, A. Auerbach, F. Li, Structural basis of receptor recognition by SARS-CoV-2, Nature 581 (7807) (2020) 221–224. doi:10.1038/s41586-020-2179-y.
URL http://www.nature.com/articles/s41586-020-2179-y

[2] S. Feola, M. Haapala, K. Peltonen, C. Capasso, B. Martins, G. Antignani, A. Federico, V. Pietiäinen, J. Chiaro, M. Feodoroff, S. Russo, A. Rannikko, M. Fusciello, S. Koskela, J. Partanen, F. Hamdan, S. M. Tähkä, E. Ylösmäki, D. Greco, M. Grönholm, T. Kekarainen, M. Eshaghi, O. L. Gurvich, S. Ylä-Herttuala, R. M. M. Branca, J. Lehtiö, T. M. Sikanen, V. Cerullo, PeptiCHIP: A Microfluidic Platform for Tumor Antigen Landscape Identification, ACS Nano 15 (10) (2021) 15992–16010. doi:10.1021/acsnano.1c04371.
URL https://pubs.acs.org/doi/10.1021/acsnano.1c04371

[3] B. Reynisson, B. Alvarez, S. Paul, B. Peters, M. Nielsen, NetMHCpan-4.1 and NetMHCIIpan-4.0: Improved predictions of MHC antigen presentation by concurrent motif deconvolution and integration of MS MHC eluted ligand data, Nucleic Acids Research 48 (W1) (2021) W449–W454. doi:10.1093/NAR/GKAA379.

[4] O. Wagih, ggseqlogo: a versatile R package for drawing sequence logos, Bioinformatics 33 (22) (2017) 3645–3647. doi:10.1093/bioinformatics/btx469.
URL https://academic.oup.com/bioinformatics/article/33/22/3645/3980251

[5] E. Eden, R. Navon, I. Steinfeld, D. Lipson, Z. Yakhini, GOrilla: a tool for discovery and visualization of enriched GO terms in ranked gene lists, BMC Bioinformatics 10 (1) (2009) 48. doi:10.1186/1471-2105-10-48.
URL https://bmcbioinformatics.biomedcentral.com/articles/10.1186/1471-2105-10-48

[6] T. Schmidt, P. Samaras, V. Dorfer, C. Panse, T. Kockmann, L. Bichmann, B. Van Puyvelde, Y. Perez-Riverol, E. W. Deutsch, B. Kuster, M. Wilhelm, Universal Spectrum Explorer: A Standalone (Web-)Application for Cross-Resource Spectrum Comparison, Journal of Proteome Research 20 (6) (2021) 3388–3394. doi:10.1021/acs.jproteome.1c00096.
