# Supplementary material for: Thunder-DDA-PASEF enables high-coverage immunopeptidomics and is boosted by MS2Rescore with MS2PIP timsTOF fragmentation prediction model
Source: Nat Commun. 2024 Mar 13;15:2288. doi: 10.1038/s41467-024-46380-y (PMC10937930; doi:10.1038/s41467-024-46380-y)
Supplement: Supplementary file 2 — Reporting Summary [file 41467_2024_46380_MOESM2_ESM.pdf]

nature portfolio

Corresponding author(s): Dr. David Gomez-Zepeda, Univ.-Prof. Dr. Stefan Tenzer

Last updated by author(s): Jan 26, 2024

Reporting Summary

Nature Portfolio wishes to improve the reproducibility of the work that we publish. This form provides structure for consistency and transparency in reporting. For further information on Nature Portfolio policies, see our Editorial Policies and the Editorial Policy Checklist.

Statistics

For all statistical analyses, confirm that the following items are present in the figure legend, table legend, main text, or Methods section.

n/a	Confirmed	
☐	☒	The exact sample size (n) for each experimental group/condition, given as a discrete number and unit of measurement
☐	☒	A statement on whether measurements were taken from distinct samples or whether the same sample was measured repeatedly
☐	☒	The statistical test(s) used AND whether they are one- or two-sided<br>*Only common tests should be described solely by name; describe more complex techniques in the Methods section.*
☐	☒	A description of all covariates tested
☐	☒	A description of any assumptions or corrections, such as tests of normality and adjustment for multiple comparisons
☐	☒	A full description of the statistical parameters including central tendency (e.g. means) or other basic estimates (e.g. regression coefficient) AND variation (e.g. standard deviation) or associated estimates of uncertainty (e.g. confidence intervals)
☐	☒	For null hypothesis testing, the test statistic (e.g. F, t, r) with confidence intervals, effect sizes, degrees of freedom and P value noted<br>*Give P values as exact values whenever suitable.*
☒	☐	For Bayesian analysis, information on the choice of priors and Markov chain Monte Carlo settings
☒	☐	For hierarchical and complex designs, identification of the appropriate level for tests and full reporting of outcomes
☐	☒	Estimates of effect sizes (e.g. Cohen's d, Pearson's r), indicating how they were calculated

Our web collection on statistics for biologists contains articles on many of the points above.

Software and code

Policy information about availability of computer code

Data collection	Data was acquired using Compass Hystar (Bruker) versions between 4 and 5.1, and timsControl versions between 3 and 4.0.5 (Bruker).
Data analysis	LC-MS immunopeptidomics data analysis was performed in PEAKS XPro (v10.6, build 20201221). MS2Rescore was used for identification rescoring. MHC-binding was predicted using NetMHCpan 4.1 and GibbsCluster 2.0 through MhcVizPipe (v0.7.9).<br>R scripts were used for data analysis: statistical difference using ggpubr (v. 0.4.0); plots were generated using ggplot2 (v. 3.4.0); Venn plots with ggvenn (v. 0.1.9); and upset plots with ggupset (v. 0.3.0).

For manuscripts utilizing custom algorithms or software that are central to the research but not yet described in published literature, software must be made available to editors and reviewers. We strongly encourage code deposition in a community repository (e.g. GitHub). See the Nature Portfolio guidelines for submitting code & software for further information.

Data

Policy information about availability of data

All manuscripts must include a data availability statement. This statement should provide the following information, where applicable:

- Accession codes, unique identifiers, or web links for publicly available datasets
- A description of any restrictions on data availability
- For clinical datasets or third party data, please ensure that the statement adheres to our policy

The protein database was composed of the UniProtKB (Swiss-Prot) reference proteomes of Homo sapiens (Taxon ID 9606, downloaded 02/Feb/2020), Epstein-Barr

virus (strain GD1, Taxon ID 10376, downloaded 06/Feb./2022), GFP from Aequorea victoria (P42212), and SARS-CoV-2 (Taxon ID 2697049, downloaded 10/March/2021), as well as the SiORF1 reported by Finkel et al. (2021), and Gordon et al. (2020), supplemented with a list of 172 possible contaminants.

The mass spectrometry immunopeptidomics and proteomics data have been deposited to the ProteomeXchange Consortium[http://proteomecentral.proteomexchange.org] via the jPOSTrepo partner repository with the dataset identifiers PXD040385 for ProteomeXchange and JPST002044 for jPOSTrepo. Data from JY immunopeptidomics used for training was previously published (Teschner et al. 2023) and can be accessed with the dataset identifiers PXD043026 for ProteomeXchange and JPST002158 for jPOSTrepo. Data from Carapito lab have been deposited to the ProteomeXchange repositories PXD046535 for HL60 immunopeptidomics, and PXD046543 for HeLa tryptic proteomics files. A list of datasets, main LC-MS parameters, and their repository locations is included as Supplementary Material S11. Protein FASTA files used for peptide and protein identification are also included in the corresponding repositories.

Accession codes:
Data from Tenzer lab:
Identifiers: PXD040385 for ProteomeXchange and JPST002044 for jPOST
URL: https://repository.jpostdb.org/preview/209791232665415e51504c9
Access key: 3041
The files of the JY immunopeptidomics data used to train MS2PIP were recently published1 and are already publicly available with the dataset identifiers PXD043026 for ProteomeXchange and JPST002158 for jPOSTrepo.
HL60 immunopeptidomics data from Carapito lab:
URL to log in: https://www.ebi.ac.uk/pride/login
Project accession: PXD046535
Username: reviewer_pxd046535@ebi.ac.uk
Password: t0tCB5Cy
HeLa tryptic proteomics data from Carapito lab:
URL to log in: https://www.ebi.ac.uk/pride/login
Project accession: PXD046543
Username: reviewer_pxd046543@ebi.ac.uk
Password: KFdT3vsx

Human research participants

Policy information about studies involving human research participants and Sex and Gender in Research.

Reporting on sex and gender	n/a
Population characteristics	n/a
Recruitment	n/a
Ethics oversight	n/a

Note that full information on the approval of the study protocol must also be provided in the manuscript.

Field-specific reporting

Please select the one below that is the best fit for your research. If you are not sure, read the appropriate sections before making your selection.

- [x] Life sciences
- [ ] Behavioural & social sciences
- [ ] Ecological, evolutionary & environmental sciences

For a reference copy of the document with all sections, see nature.com/documents/nr-reporting-summary-flat.pdf

Life sciences study design

All studies must disclose on these points even when the disclosure is negative.

Sample size	Pooled samples were used for method development. To enable statistical analysis, aliquots were analyzed in three LC-MS injection replicates in most of the experiments. The data presented in Figure 4, showing multiple sample types analyzed with four distinct methods, was acquired in technical duplicates due to limited sample and instrument availability at the time of the analysis. Thus, statistics were not performed in Figure 4, but the qualitative evaluation is sufficient to support the conclusions.<br>For the JY and Raji data set, three cultures of each WT cell line (JY_WT and Raji_WT) and two different cultures of each transfected cell line (JY_S1, JY_S2, Raji_S1, and Raji_S2) were processed separately, and each sample analyzed in three LC-MS injection replicates. The sample size was determined by sample and instrument availability at the time of the experiment. Here, the objective was to profile the HLAI immunopeptidome of the cell lines qualitatively, and thus, no statistical analysis was performed.
Data exclusions	No data were excluded

Replication	Method optimization was validated by repeating the experiment two times. Identification of spike immunopeptides reproducibility was ensured by the replicates mentioned in the sample size section. In addition, the identification confidence (score) and reproducibility are reported in the manuscript and supplementary materials.
Randomization	Method comparison (Fig. 1 to 6): To ensure that method comparisons were not affected by sample or instrument variability, aliquots of the same pooled sample were analyzed sequentially by intercalating the diverse methods whenever possible. Randomization was not applied in this case since it would have negatively affected the results.<br>JY and Raji cells transfected to express the spike protein (Fig. 7 and 8): The distinct cell lines were injected in separate batches to minimize the risk of carry-over between samples. Wild-type and transfected samples were injected in a block-randomized way, including samples of each kind in each technical replicate batch.
Blinding	Blinding was not done in this study since knowing the sample and method used for the analysis was necessary to perform all the comparisons.

Reporting for specific materials, systems and methods

We require information from authors about some types of materials, experimental systems and methods used in many studies. Here, indicate whether each material, system or method listed is relevant to your study. If you are not sure if a list item applies to your research, read the appropriate section before selecting a response.

Materials & experimental systems

n/a	Involved in the study
☐	☒ Antibodies
☐	☒ Eukaryotic cell lines
☒	☐ Palaeontology and archaeology
☒	☐ Animals and other organisms
☒	☐ Clinical data
☒	☐ Dual use research of concern

Methods

n/a	Involved in the study
☒	☐ ChIP-seq
☐	☒ Flow cytometry
☒	☐ MRI-based neuroimaging

Antibodies

Antibodies used	Tenzer Lab: The anti-panHLA Class I antibody W6/32 (anti-HLA-A, -B, -C) was purchased from Hoelzel-biotech, and produced by Leinco Technologies (ref. H263)<br>Carapito Lab: he HB-95 hybridoma producing anti-panHLA Class I antibody W6/32 was purchased from ATCC and cultured in Panserin 401 serum free medium (Pan Biotech). The purification of the antibody was done with the NGC Chromatography System (Biorad) using a HiTrap Protein G HP 1 mL column (Amersham Pharmacia).
Validation	Commercial, and commonly used for similar studies.<br>Specificity: Clone W6/32 recognizes the human MHC class I molecules HLA-A, -B, and -C.<br>Reactive Species: Baboon, chimpanzee, cynomolgus monkey, feline, bovine, human.<br>Host species: Mouse<br>Purity: ≥95% monomer by analytical SEC and SDS Page<br>References:<br>https://www.leinco.com/p/anti-human-hla-a-b-c-mhc-class-ipurified-functional-grade-gold/<br>Nelde, A., Kowalewski, D. J. & Stevanović, S. Purification and Identification of Naturally Presented MHC921<br>Class I and II Ligands, 123–136 (Springer New York, New York, NY, 2019). URL https://doi.org/10.922<br>1007/978-1-4939-9450-2{_}10http://link.springer.com/10.1007/978-1-4939-9450-2{_}10.

Eukaryotic cell lines

Policy information about cell lines and Sex and Gender in Research

Cell line source(s)	The human B lymphoblastoid cell line JY and HL60 cell line were purchased from ATCC, and the human Burkitt lymphoma cell line Raji was obtained by the DSMZ-German Collection of Microorganisms and Cell Cultures. HeLa (CVCL_0030) whole cell pellets were purchased from Ipracell (Belgium, CC-01-10-50). Human plasma was purchased from Zen-Bio (USA, SER-SPL, fresh frozen, in 4% sodium citrate). The human melanoma cell line SK-MEL-37 was purchased from sigma-aldrich (SCC262).
Authentication	Transfected G418-resistant and eGFP-expressing cells were selected by three rounds of screening using a FACS Aria
Mycoplasma contamination	All cell lines tested negative for mycoplasma contamination.
Commonly misidentified lines (See ICLAC register)	No commonly misidentified cell lines were used in the study.

Flow Cytometry

Plots

Confirm that:

- [ ] The axis labels state the marker and fluorochrome used (e.g. CD4-FITC).
- [ ] The axis scales are clearly visible. Include numbers along axes only for bottom left plot of group (a 'group' is an analysis of identical markers).
- [ ] All plots are contour plots with outliers or pseudocolor plots.
- [ ] A numerical value for number of cells or percentage (with statistics) is provided.

Methodology

Sample preparation	*Describe the sample preparation, detailing the biological source of the cells and any tissue processing steps used.*
Instrument	*Identify the instrument used for data collection, specifying make and model number.*
Software	*Describe the software used to collect and analyze the flow cytometry data. For custom code that has been deposited into a community repository, provide accession details.*
Cell population abundance	*Describe the abundance of the relevant cell populations within post-sort fractions, providing details on the purity of the samples and how it was determined.*
Gating strategy	*Describe the gating strategy used for all relevant experiments, specifying the preliminary FSC/SSC gates of the starting cell population, indicating where boundaries between "positive" and "negative" staining cell populations are defined.*

- [ ] Tick this box to confirm that a figure exemplifying the gating strategy is provided in the Supplementary Information.
