# Supplementary material for: Thunder-DDA-PASEF enables high-coverage immunopeptidomics and is boosted by MS2Rescore with MS2PIP timsTOF fragmentation prediction model
Source: Nat Commun. 2024 Mar 13;15:2288. doi: 10.1038/s41467-024-46380-y (PMC10937930; doi:10.1038/s41467-024-46380-y)
Supplement: Supplementary file 4 — Description of Additional Supplementary Files [file 41467_2024_46380_MOESM4_ESM.pdf]

Supplementary Data 1. Extended Method Settings

Excel file with detailed settings of the methods used, containing the following information:

- experiments: Summary of the experiments performed, indicating the parameters that were modified, and the figures or Supplementary Data corresponding to the data set
- ms_thunder: Optimized Thunder-DDA-PASEF method for HLA class I peptide ligands (HLAIps)
- isolation_polygon_thunder Coordinates: indicating the edges of the Thunder HLAIp-tailored isolation polygon for fragmentation
- LC_47min: 47 min LC gradient used for initial tests
- LC_110min: 110 min LC gradient used for cycle time optimization and final samples
- PEAKS_XPro: Parameters used for peptide identification in PEAKS Xpro
- MS^2Rescore: Parameters used for identification rescoring in MS^2Rescore
- MhcVizPipe: Parameters used for HLA-binding prediction with NetMHCpan-4.1 and GibbsCluster-2.0 via MhcVizPipe

Supplementary Data 2. Thunder-DDA-PASEF method for timsTOF Pro-2 (timsControl v3)

Compressed Bruker method ".m" folder containing the optimized Thunder-DDA-PASEF MS method for timsTOF Pro instruments. Please not that it was created in timsControl v3 and it may not be compatible with future versions, or other types of timsTOF instruments.

Supplementary Data 3. Peptides identified using 100ms_None, 100ms_Thunder, 300ms_None, 300ms_Thunder in JY, HeLa, SK-MEL-37, and human plasma

Output from PEAKS, compiled and formatted results in csv format, compressed file.

Supplementary Data 4. HLA class I binding prediction for the 8 to 13-mers identified using 100ms_None, 100ms_Thunder, 300ms_None, 300ms_Thunder in JY, HeLa, SK-MEL-37, and human plasma

Binding prediction to the JY HLA alleles for the 8 to 13-mers reported in Supplementary Data S3. Output from MhcVizPipe [1] with predictions using NetMHCpan-4.1 [2]. The compressed file contains the compiled and formatted results in csv format.

Supplementary Data 5. Peptides identified in JY and Raji cells WT and transfected to express segments of the SARS-CoV-2 spike protein

Output from PEAKS, compiled and formatted results in csv format, compressed file.

Supplementary Data 6. HLA class I binding prediction for the 8 to 13-mers identified in JY and Raji cells, with matched HLA alleles

Binding prediction to the JY or Raji HLA alleles, respectively to the cell line where the peptides were identified, for the 8 to 13-mers reported in Supplementary Data S5. Output from MhcVizPipe [1] with predictions using NetMHCpan-4.1 [2]. The compressed file contains the compiled and formatted results in csv format.

Supplementary Data 7. GO enrichment analysis of JY and Raji common proteins covered by HLA class I ligands

Excel file containing the results of the (GO) enrichment analysis using GOrilla [3] for the proteins identified in the immunopeptidome (HLA class I ligands) of only JY, only Raji or both cell lines (tabs starting with "jy", "raji", or "jy_raji", respectively). The results include the three different ontologies: function, component, and process.

Supplementary Data 8. Spike immunopeptidome

Excel file with the results for the spike peptides identified in JY and Raji cells.

Content: The tabs are organized as follows

- ms_mhcpred_all_alleles: merged MS results and HLA-binding predictions from NetMHCpan-4.1
- ms_mhcpred_merged_minrank: merged MS results and HLA-binding predictions from NetMHCpan-4.1, considering only the HLA allele with the lowest EL_rank
- peptides_ms_summary: MS results summarized per peptide and Cell Line
- validation: summary of the validation of MS identifications compared to synthetic peptides or PROSIT predicted spectra
- peptides_ms: MS results, spike peptides identified in JY or Raji cells
- iedb: Data downloaded from the Immune Epitope Database (IEDB) the 18 December 2022
- mhcpred: HLA-binding predictions from NetMHCpan-4.1, via MhcVizPipe

Supplementary Data 9. MS2PIP timsTOF prediction hyperoptimization

The hyperoptimization rounds for the MS2PIP timsTOF prediction models were logged to weights and biases. The interactive version is available in the link below. A pdf-printed version is attached as Supplementary Data S10.

https://wandb.ai/arthur_declercq/Final%20timstof%20model%20training/reports/MS-PIP-timsTOF-prediction-models--Vmlldzo1NjQxNTMw.

Supplementary Data 10. List of datasets and their repository locations

The csv file includes a list of datasets with the main LC-MS parameters for each experiment, and their repository accession for ProteomeXchange and jPOSTrep.

References

[1] K. A. Kovalchik, Q. Ma, L. Wessling, F. Saab, J. D. Duquette, P. Kubiniok, D. J. Hamelin, P. Faridi, C. Li, A. W. Purcell, A. Jang, E. Paramithiotis, M. Tognetti, L. Reiter, R. Bruderer, J. Lanoix, É. Bonneil, M. Courcelles, P. Thibault, E. Caron, I. Sirois, MhcVizPipe: A Quality Control Software for Rapid Assessment of Small- To Large-Scale Immunopeptidome Datasets, Molecular and Cellular Proteomics 21 (1) (2022) 0–14. doi:10.1016/j.mcpro.2021.100178.

[2] B. Reynisson, B. Alvarez, S. Paul, B. Peters, M. Nielsen, NetMHCpan-4.1 and NetMHCIIpan-4.0: Improved predictions of MHC antigen presentation by concurrent motif deconvolution and integration of MS MHC eluted ligand data, Nucleic Acids Research 48 (W1) (2021) W449–W454. doi:10.1093/NAR/GKAA379.

[3] E. Eden, R. Navon, I. Steinfeld, D. Lipson, Z. Yakhini, GOrilla: a tool for discovery and visualization of enriched GO terms in ranked gene lists, BMC Bioinformatics 10 (1) (2009) 48. doi:10.1186/1471-2105-10-48.
URL https://bmcbioinformatics.biomedcentral.com/articles/10.1186/1471-2105-10-48
