# Supplementary material for: Thunder-DDA-PASEF enables high-coverage immunopeptidomics and is boosted by MS2Rescore with MS2PIP timsTOF fragmentation prediction model
Source: Nat Commun. 2024 Mar 13;15:2288. doi: 10.1038/s41467-024-46380-y (PMC10937930; doi:10.1038/s41467-024-46380-y)
Supplement: Supplementary file 13 — Supplementary Data 9 [file 41467_2024_46380_MOESM13_ESM.pdf]

Share Comment Star

MS^2PIP timsTOF prediction models

Newly trained peak intensity prediction models for data from timsTOF instruments

Arthur Declercq

train_rmse_avg and max_leaves v. test_rmse_avg

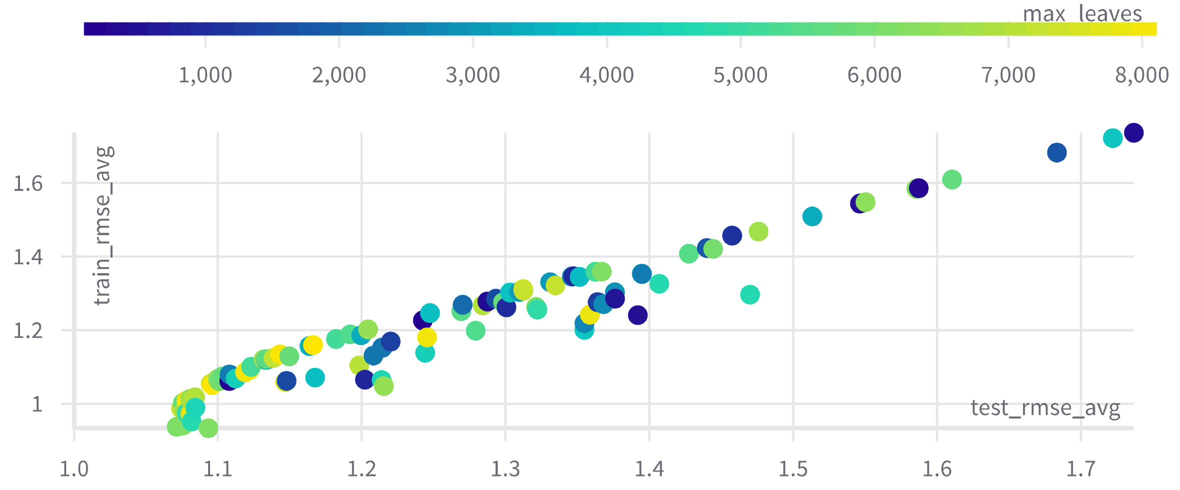

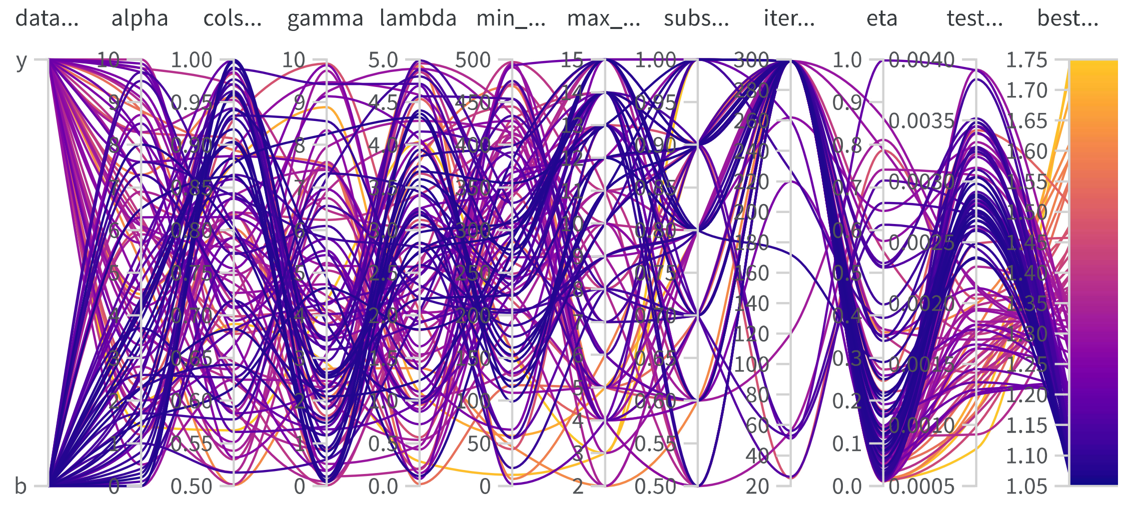

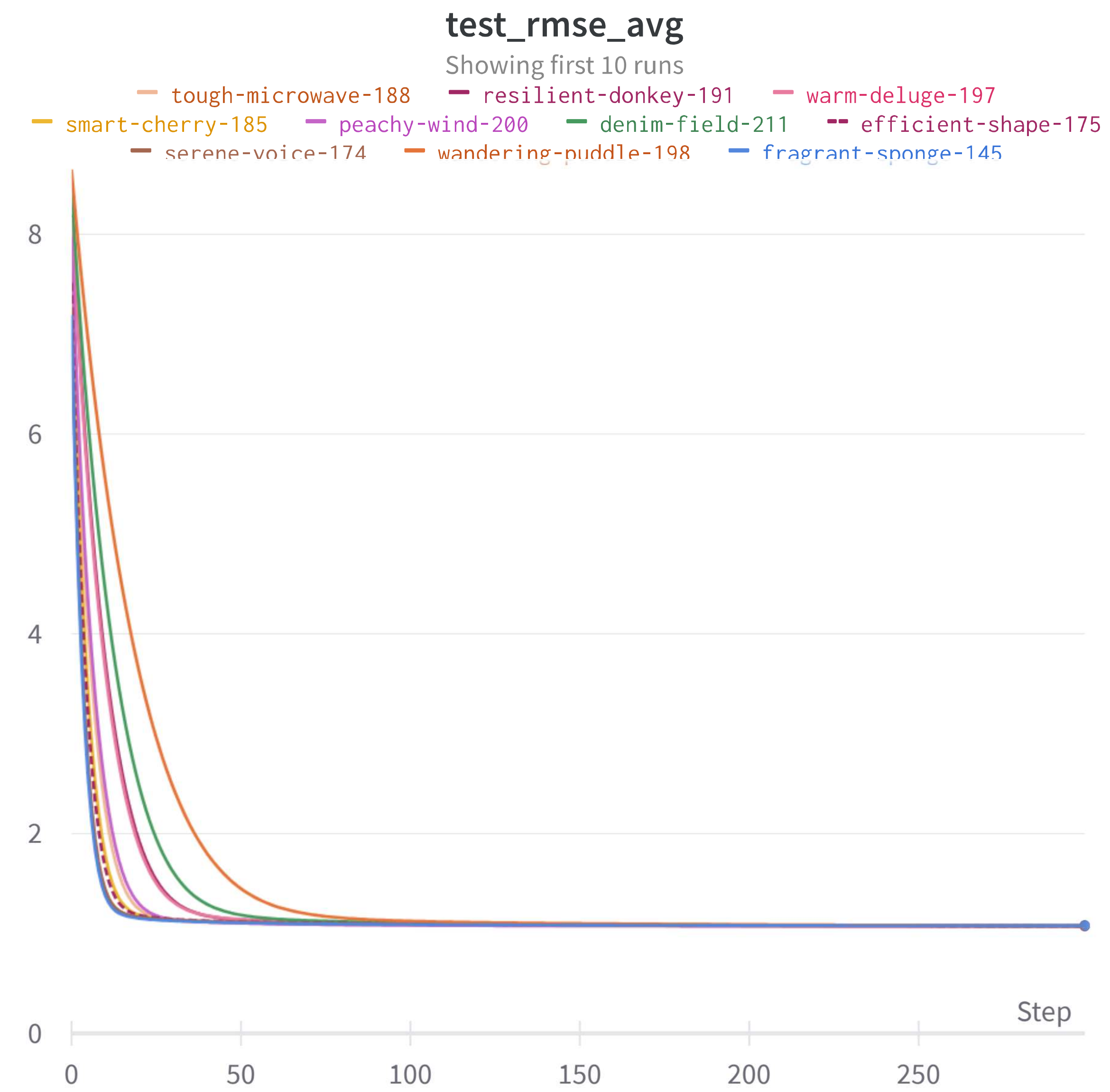

Parameter importance with respect to best_loss

Search

Parameters

1-9 of 9

Config parameter	Importance	Correlation
max_depth		
min_child_weight		
max_leaves		
eta		

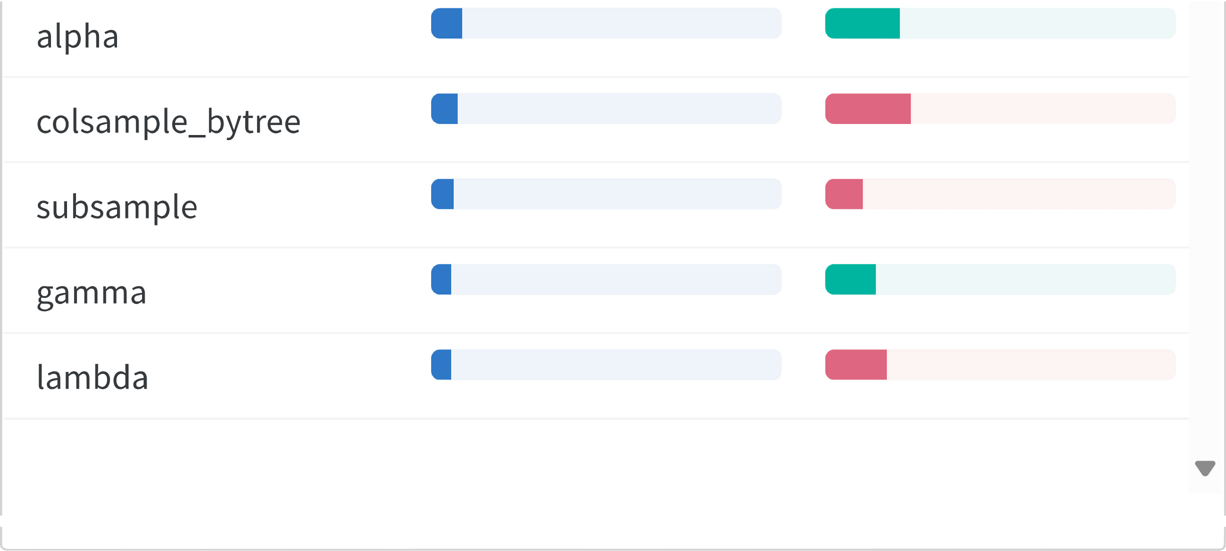

Created with ❤️ on Weights & Biases.

https://wandb.ai/arthur_declercq/Final%20timstof%20model%20training/reports/MS-PIP-timsTOF-prediction-models--Vmlldzo1NjQxNTMw
